# Characterization of terminal-ileal and colonic Crohn’s disease in treatment-naïve paediatric patients based on transcriptomic profile using logistic regression

**DOI:** 10.1186/s12967-021-02909-z

**Published:** 2021-06-07

**Authors:** Ilkyu Park, Jaeeun Jung, Sugi Lee, Kunhyang Park, Jea-Woon Ryu, Mi-Young Son, Hyun-Soo Cho, Dae-Soo Kim

**Affiliations:** 1 Department of Bioinformatics, KRIBB School of Bioscience, Korea University of Science and Technology (UST), 217 Gajeong-ro, Yuseong-gu, Daejeon, Korea; 2 Department of Environmental Disease Research Center, Korea Research Institute of Bioscience & Biotechnology (KRIBB), 125 Gwahak-ro, Yuseong-gu, Daejeon, 34141 Korea; 3 Department of Core Facility Management Center, Korea Research Institute of Bioscience & Biotechnology (KRIBB), 125 Gwahak-ro, Yuseong-gu, Daejeon, Korea; 4 Department of Rare Disease Research Center, Korea Research Institute of Bioscience & Biotechnology (KRIBB), 125 Gwahak-ro, Yuseong-gu, Daejeon, Korea; 5 Department of Stem Cell Convergence Research Center, Korea Research Institute of Bioscience & Biotechnology (KRIBB), 125 Gwahak-ro, Yuseong-gu, Daejeon, Korea

**Keywords:** Crohn’s disease, Colonic CD, Terminal-ileal CD, Paediatric patients, Transcriptomic profile, Logistic regression

## Abstract

**Background:**

Inflammatory bowel disease (IBD) is a chronic and idiopathic inflammatory disorder of the gastrointestinal tract and comprises ulcerative colitis (UC) and Crohn’s disease (CD). Crohn’s disease can affect any part of the gastrointestinal tract, but mainly the terminal ileum and colon. In the present study, we aimed to characterize terminal-ileal CD (ICD) and colonic CD (CCD) at the molecular level, which might enable a more optimized approach for the clinical care and scientific research of CD.

**Methods:**

We analyzed differentially expressed genes in samples from 23 treatment-naïve paediatric patients with CD and 25 non-IBD controls, and compared the data with previously published RNA-Seq data using multi-statistical tests and confidence intervals. We implemented functional profiling and proposed statistical methods for feature selection using a logistic regression model to identify genes that are highly associated in ICD or CCD. We also validated our final candidate genes in independent paediatric and adult cohorts.

**Results:**

We identified 550 genes specifically expressed in patients with CD compared with those in healthy controls (p < 0.05). Among these DEGs, 240 from patients with CCD were mainly involved in mitochondrial dysfunction, whereas 310 from patients with ICD were enriched in the ileum functions such as digestion, absorption, and metabolism. To choose the most effective gene set, we selected the most powerful genes (*p*-value ≤ 0.05, accuracy ≥ 0.8, and AUC ≥ 0.8) using logistic regression. Consequently, 33 genes were identified as useful for discriminating CD location; the accuracy and AUC were 0.86 and 0.83, respectively. We then validated the 33 genes with data from another independent paediatric cohort (accuracy = 0.93, AUC = 0.92) and adult cohort (accuracy = 0.88, AUC = 0.72).

**Conclusions:**

In summary, we identified DEGs that are specifically expressed in CCD and ICD compared with those in healthy controls and patients with UC. Based on the feature selection analysis, 33 genes were identified as useful for discriminating CCD and ICD with high accuracy and AUC, for not only paediatric patients but also independent cohorts. We propose that our approach and the final gene set are useful for the molecular classification of patients with CD, and it could be beneficial in treatments based on disease location.

**Supplementary Information:**

The online version contains supplementary material available at 10.1186/s12967-021-02909-z.

## Introduction

The incidence and prevalence of inflammatory bowel disease (IBD) are increasing worldwide and is emerging as a global disease [1]. According to current studies, IBD including Crohn’s disease (CD) and ulcerative colitis (UC) is caused by complex interactions between genetic backgrounds and environmental signals, leading to chronic inflammation of the gastrointestinal (GI) tract [2, 3]. Particularly, CD causes inadequate chronic activation of the mucosal immune system resulting from an aberrant immune response to enteric microbiota throughout the GI tract [4, 5]. Inflammation in the digestive tract impairs food digestion and nutrient absorption. Recently, several studies reported a potential relation between CD and other diseases such as Parkinson’s disease (PD) and non-alcoholic fatty liver disease (NAFLD). In addition, a meta-analysis of four studies comprising approximately 100,000 patients with IBD and millions of controls also highlighted the PD risk in the IBD population; the overall risk of PD in IBD patients was remarkably higher than in controls, and patients with CD had a 28% increased risk of PD [6]. Similarly to PD, NAFLD was detected in up to 33.6% of patients with IBD and its prevalence in patients with CD was reported in cross-sectional studies as 6.2–40% [7–10].

CD manifests in various locations and its symptoms depend on the severity of inflammation, but generally reaches two major tissue locations: the colon and end of the small intestine [11]. Although diagnosing these two tissue sites by colonoscopy is comparatively easy, cases in many children and adults patients remain “unclassified” despite disease progression [12]. The distinction between these two CD locations is critical for correct clinic treatment [13]. Current clinical treatment is limited to the blockade of inflammatory mediators [14]. However, as CD symptoms vary according to the onset location and patient characteristics, the diagnosis of colonic CD (CCD) and terminal-ileal CD (ICD) should be standardized to allow for the development of more personalized disease treatments and management. Many previous studies investigated the differentiation between CD and UC in patients with IBD to understand the mechanism underlying disease pathogenesis; however, few studies have focused on discriminating CCD and ICD based on locations because of its complex genetic traits, with genetic heterogeneity and incomplete phenotype penetrance [12]. Although multi-omics profiling approaches have been attempted [15], the molecular pathology of CD is not well-understood because of difficulties in classifying CD locations based on gene expression differences.

Although CD can occur at any age, 20–30% of patients are diagnosed in childhood [16]. It remains unclear how environmental factors lead to development of the IBD phenotype and its genetic heterogeneity and subsequent evolution. Therefore, the study of paediatric-onset CD is considered an essential need in elaborating a precise strategy for CD diagnosis and treatment.

In this study, we examined treatment-naïve paediatric patients with CD without any potential impacts on the disease. We employed distinct statistical techniques to assess variations in the RNA levels of two major groups of individuals with well-characterized CCD and ICD, as well as of non-IBD controls. In contrast to previous studies, we used confidence intervals (CIs) rather than fold-changes to identify specific genes differentially and specifically expressed in each disease subtype [17]. We also analyzed the functional commonalities of each type of differentially expressed gene (DEG) and characterized CCD and ICD according to their expression patterns. Moreover, UC patients were used to filter UC characteristics from the genes, and then a logistic regression (LR) method was used to select the features that discriminate CCD and ICD in paediatric patients. Finally, these candidate genes were applied to independent paediatric and adult cohorts to validate their classification power in all groups of patients, regardless of age.

## Results

### Patient population

**Fig. 1:**
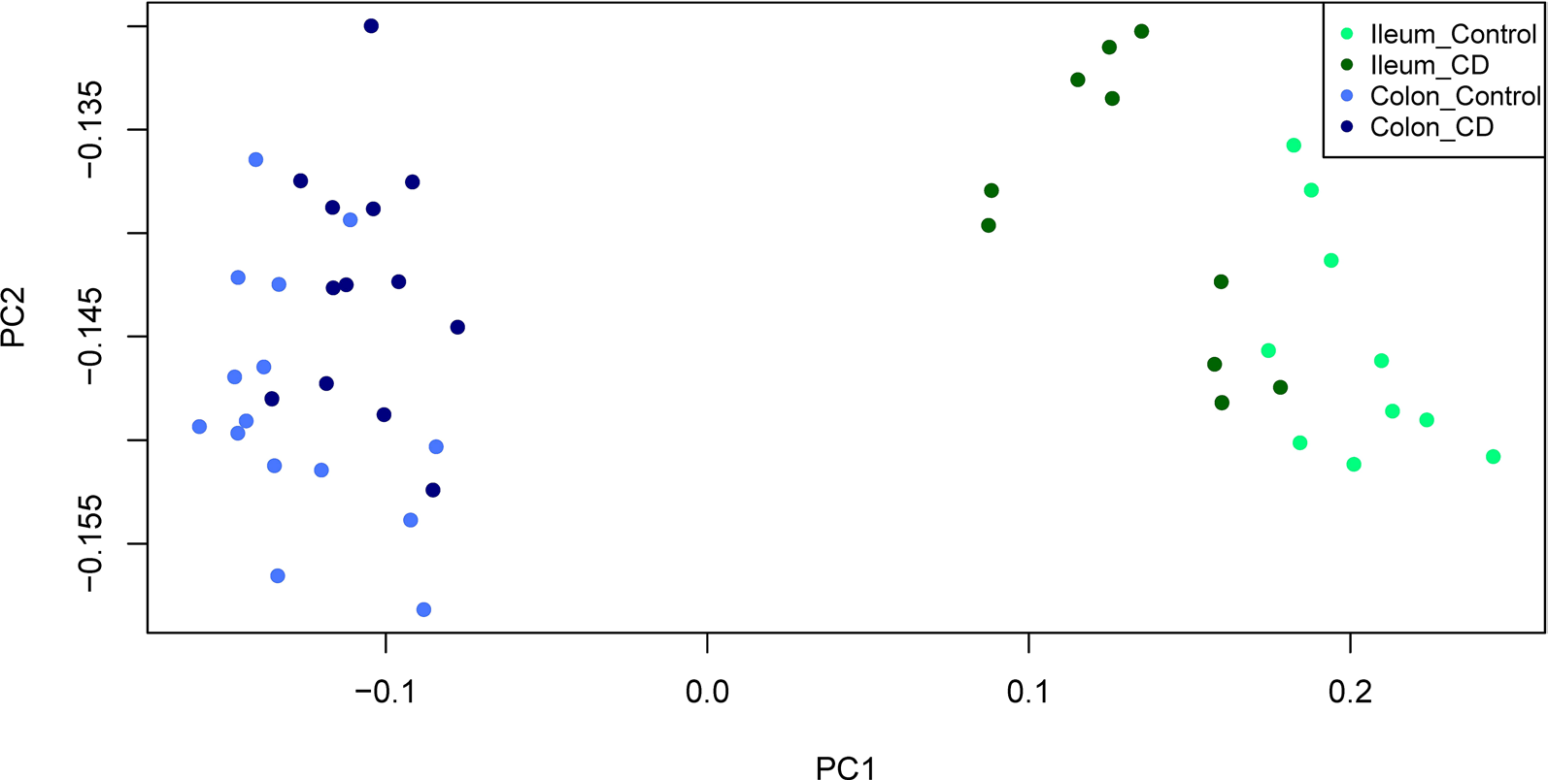
Principal component analysis (PCA) of newly diagnosed patients with paediatric CD. Unsupervised PCA analysis revealed differences according to diagnosis and gut segment; colon control (royal blue) vs. ileum control (spring green) and colon CD (navy blue) vs. ileum CD (dark green). *CD* Crohn’s disease

As some factors influencing the development of CD may affect disease heterogeneity, this study was conducted to guide the genetic classification of children and adolescents with CD by studying the pubertal stage of patients newly diagnosed with CD. RNA-Seq data of 23 paediatric patients with CD and 25 children without IBD (controls) were analyzed (Additional file 2: Table S1). Samples were collected at the time of CD onset and all patients were younger than 15 years old and were mostly male, but sex of healthy controls was not biased. Two colon CD samples were removed from analysis after being classified as outliers by an initial principal component analysis (PCA) and correlation between samples (Additional file 1: Fig. S1). The PCA revealed significant differences between the ileum and colon samples in the CD and control groups; in each tissue type, the PCA differed between CD and controls (Fig. 1 and Additional file 1: Fig. S2). Therefore, the differences between the CCD and ICD may enable discrimination of the disease in each tissue type.

### Signature of treatment-naïve CCD and ICD

We attempted to identify signatures for distinguishing CD of the colon and terminal ileum using gene expression data. Using RNA-Seq data obtained from Sequence Read Archive (SRA) of NCBI, we calculated fragments per kilobase million values of protein-coding genes (see “Materials and methods” section for more detail). We performed the Levene’s test and Shapiro test with *t* test and Wilcoxon signed-rank test to identify DEGs from four types of pairwise comparisons between CCD and colon controls (NMC), between ICD and ileum controls (NMI), between CCD and ICD, and between NMC and NMI. According to these four comparisons, 4559, 4192, 5058, and 7838 genes were differentially expressed (*p*-value ≤ 0.05), respectively. To identify genes with differential expression from the comparison set, we calculate the confidence intervals (CIs). The CI determines the interval of expression between the comparison set and selects a gene that does not overlap each other (Additional file 1: Fig. S3) [17]. After identifying DEGs (*p*-value ≤ 0.05), CIs were applied to more precisely differentiate the two conditions (CI 95%, Additional file 2: Table S2-1 and S2-2). Each condition was used to calculate the highest confidence interval (HCI) and lowest confidence interval (LCI), and genes were chosen when one LCI was higher than the other HCI. These steps were performed using all colon samples across all ileum samples first, and then implemented in each pairwise comparison. For instance, according to the CI, each condition had an estimated 95% CI area for every gene, and those with 95% CI area that did not overlap with another area were selected (Additional file 1: Fig. S3) [17]. Through this process, false-positive genes were eventually be filtered out and only true-positive genes were preserved. According to above four comparisons, 928, 915, 2808, and 5056 genes (one LCI > other HCI, CI 95%) were obtained, respectively.

**Fig. 2:**
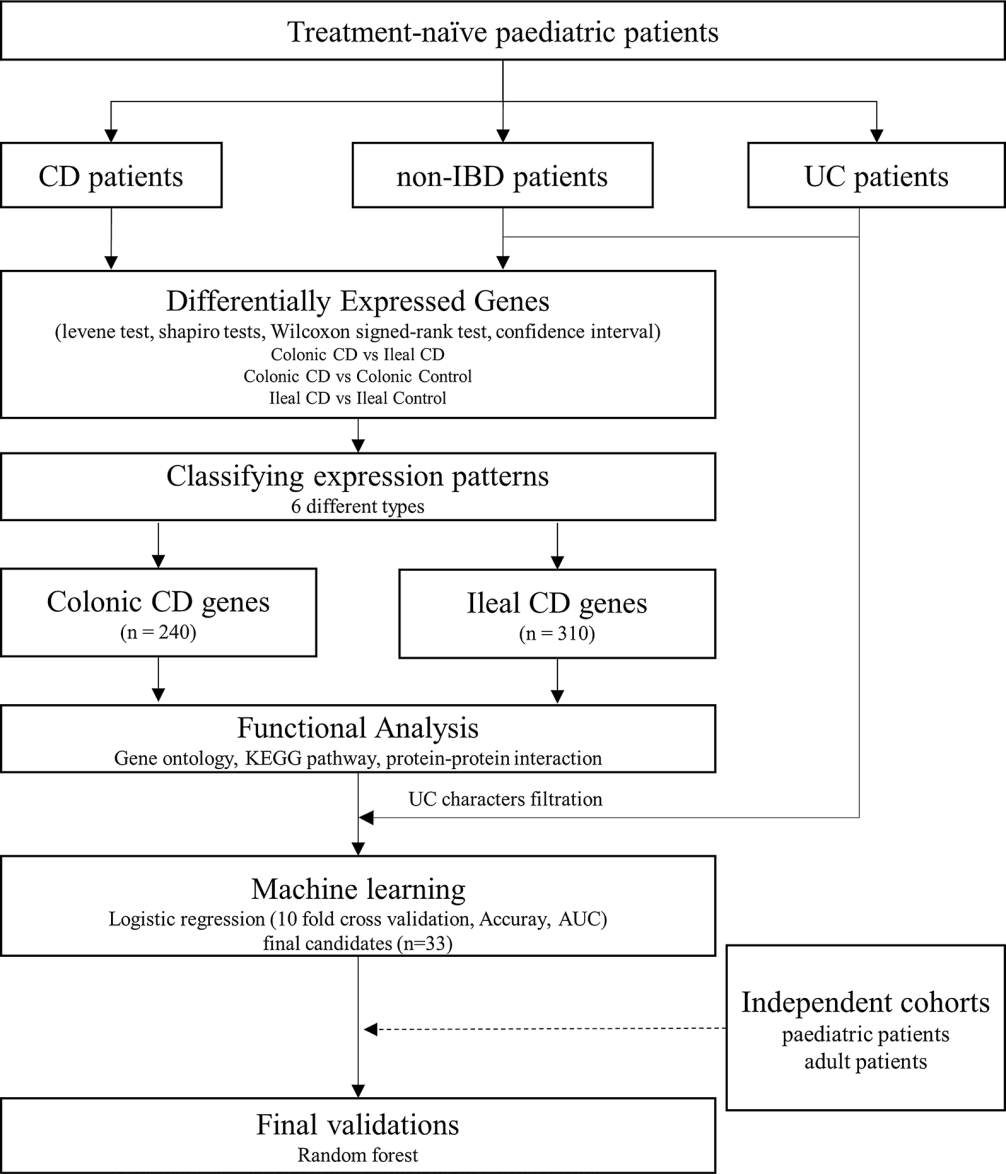
Overview of the study design. Schematic representation of this research. *CD* Crohn’s disease, *UC* ulcerative colitis, *AUC* area under curve

**Fig. 3:**
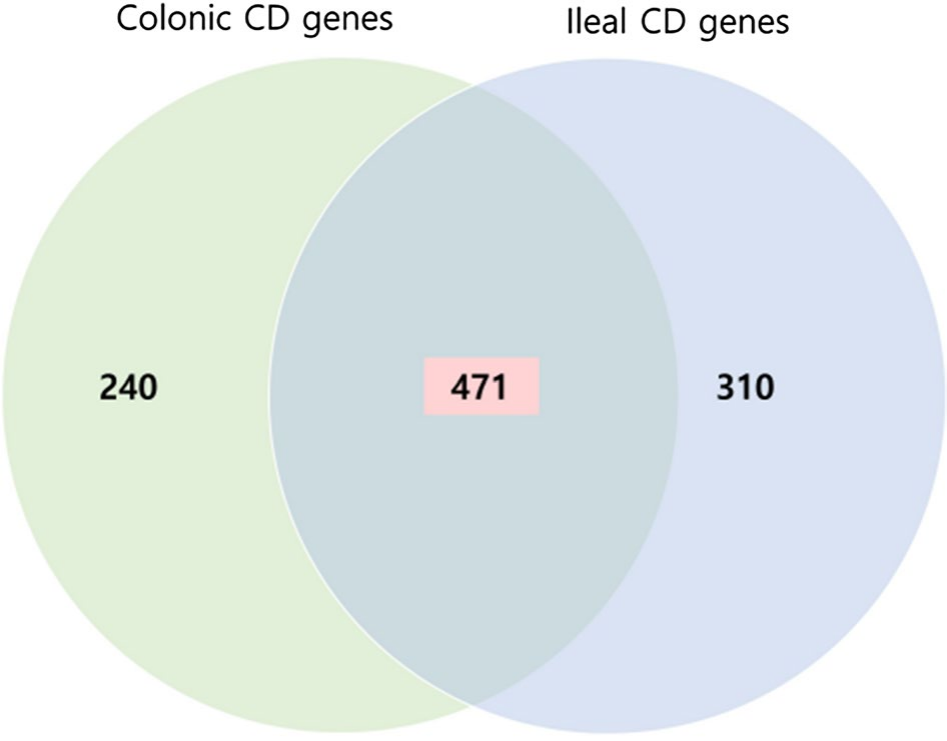
A Venn diagram illustrating differentially expressed genes in each tissue type. CCGs [n = 240] and ICGs [n = 310] were obtained in mucosal biopsies samples. Shaded area indicates CD common DEGs [n = 471] regardless of tissue type; *CD* Crohn’s disease

After applying the CI method, the final genes were selected according to the filtering process (Fig. 2). We drew a Venn diagram with four different comparisons and examined all possible number of cases to distinguish CCD and ICD (Additional file 1: Fig. S4). First, the genes differentially expressed between CCD and NMC and between ICD and NMI were selected among the DEGs between CCD and ICD. Six cases represented the differences between CCD and ICD, intersection of all comparisons (91 genes), intersection of CCD vs. ICD and CCD vs. NMC (30 genes), intersection of CCD vs. ICD and CCD vs. NMC and ICD vs. NMI (7 genes), intersection of CCD vs. ICD and ICD vs. NMI (31 genes), intersection of CCD vs. ICD and ICD vs. NMI and NMC vs. NMI (437 genes), and intersection of CCD vs. ICD and CCD vs. NMC and NMC vs. NMI (319 genes). Using these genes, we performed the Wilcoxon signed-rank test and Shapiro test with *t*-test among CCD, NMC, ICD, and NMI and recalculated *p*-values for each gene to identify different genes useful for characterizing tissue-specific CD. In defining CCD features, there was a significant distinction between CCD and ICD and between CCD and NMC (*p*-value ≤ 0.05) and there was no disparity between ICD and NMI (*p*-value > 0.1) features. We identified genes regulated in only CCD and not in ICD. When ICD features were defined, DEGs between CCD and ICD and ICD and NMI (*p*-value ≤ 0.05) and those showing no differences between CCD and NMC (*p*-value > 0.1) were extracted. As a result, two types of tissue-specific genes were used to characterize the CD locations: CCD genes (CCGs) and ICD genes (ICGs). The total numbers of CCGs and ICGs common to CD were 240, 310, and 471, respectively (Fig. 3). We compared 310 ICGs and known ICD genes; 119 genes were also identified in a previous study (1281 ileal signature genes of Haberman et al. [3] and 534 ileum-like genes of Weiser et al. [18]) and 92 genes were observed in all studies including our results (Additional file 1: Fig. S5).

**Fig. 4:**
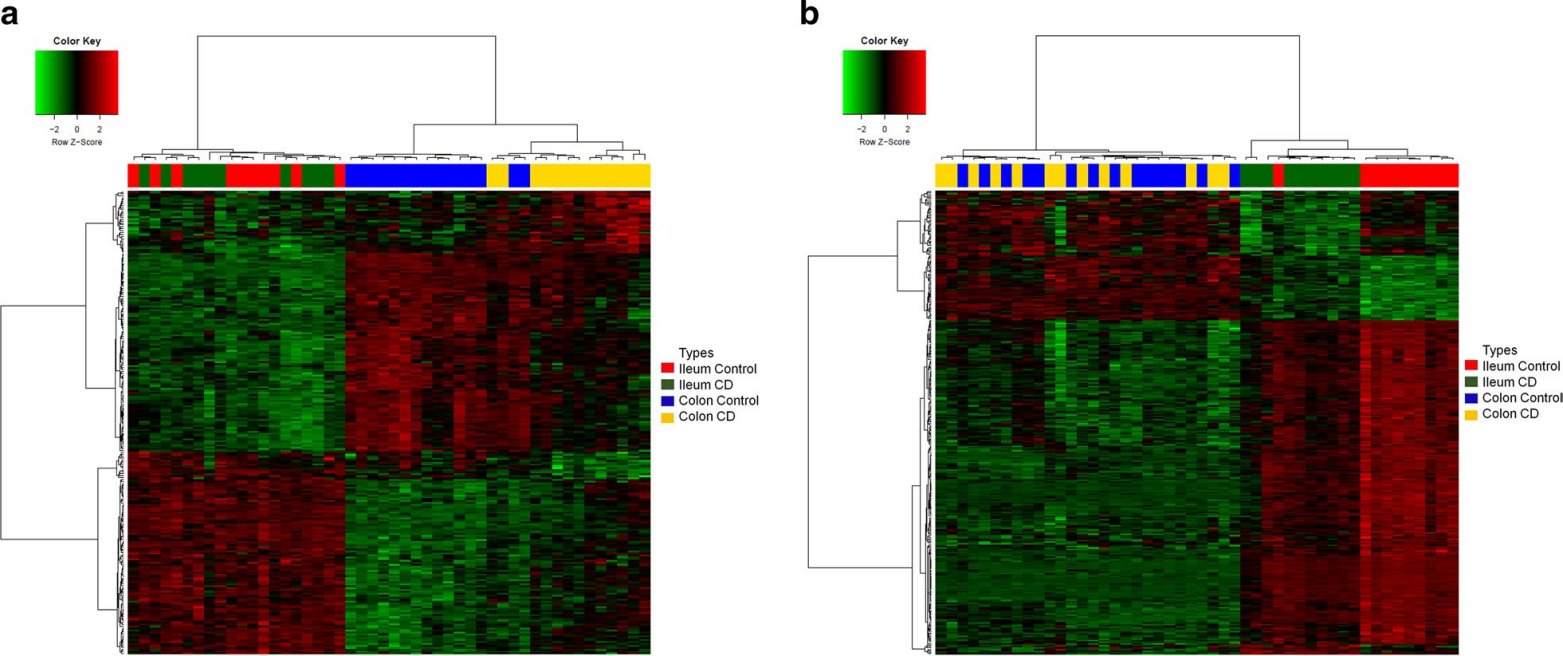
Samples from treatment-naïve paediatric patients with CD show molecular differences. Hierarchical clustering and heatmaps of RNA-Seq data of CCGs and ICGs with six different expression types. Samples are color-coded in the top bar according to the sample types and diagnosis [CD, control] and genes are color-coded in the left sidebar according to their expression types. **a** A heatmap of CCGs. **b** A heatmap of ICGs. *CD* Crohn’s disease

After defining two major types of CCD and ICD-specific genes, CCGs and ICGs (n = 220, 293), we analyzed the dataset containing these genes to verify whether they enabled characterization by tissue type compared to controls by drawing a heatmap (Additional file 1: Fig. S6). Together, all CCD and ICD-specific genes and the four sample types were classified according to their characteristics, supporting the existence of two molecularly distinct tissue type of CD. Based on hierarchical clustering analysis of these genes, each CD tissue type was clearly divided into three groups considering patients with CD and controls (Fig. 4). As shown in Fig. 4a, subjects were clearly divided into three groups: patients with ileum CD and controls, colon controls, and patients with colon CD. This revealed that CCGs could discriminate not only the colon from the ileum, but also the disease from controls. Figure 4b also shows that samples were separated into three groups: patients with colon CD and controls, ileum controls, and patients with ileum CD. Like CCGs, ICGs could differentiate colon CD from colon controls as well as colon samples from ileum samples. Therefore, the DEGs of each CCD and ICD enabled the discrimination of samples by both tissue and disease type.

As shown in Fig. 4, different gene expression patterns were observed in both CCGs and ICGs. Therefore, we examined whether CCD and ICD-specific genes showed characteristic expression patterns. As a result, six types (from A–F) of expression patterns were identified in each gene group (Table 1, Additional file 1: Fig. S7). Among the colonic type, types A and B were upregulated in both CCD and NMC compared to in ileum samples. Type A was upregulated in CCD compared to in NMC (n = 16). Type B was downregulated in CCD compared to in NMC (n = 103). Types C and D were downregulated in both CCD and NMC compared to in ileum samples. Type C was upregulated in CCD compared to in NMC (n = 91). Type D was downregulated in CCD compared to in NMC (n = 6). The genes of four types from A to D contained DEGs of normal tissues. Nevertheless, significant differences were detected between CCD and NMC and between CCD and ICD. This indicates that the tissue-specific genes also play a role in the pathogenic process by regulating their expression. In contrast, types E and F showed no differences among the NMC, NMI, and ICD samples. Type E consisted of CCD upregulated genes (n = 16) and type F consisted of CCD downregulated genes (n = 8). The genes from these two types were differentially regulated in response to the pathogenic mechanisms of CD in the colon regardless of their tissue-specific features. For the results in the ileal type, see Table 1. The complete list of DEGs in each CCD and ICD among the six different types is shown in Additional file 2: Tables S3, S4.

**Table 1:**
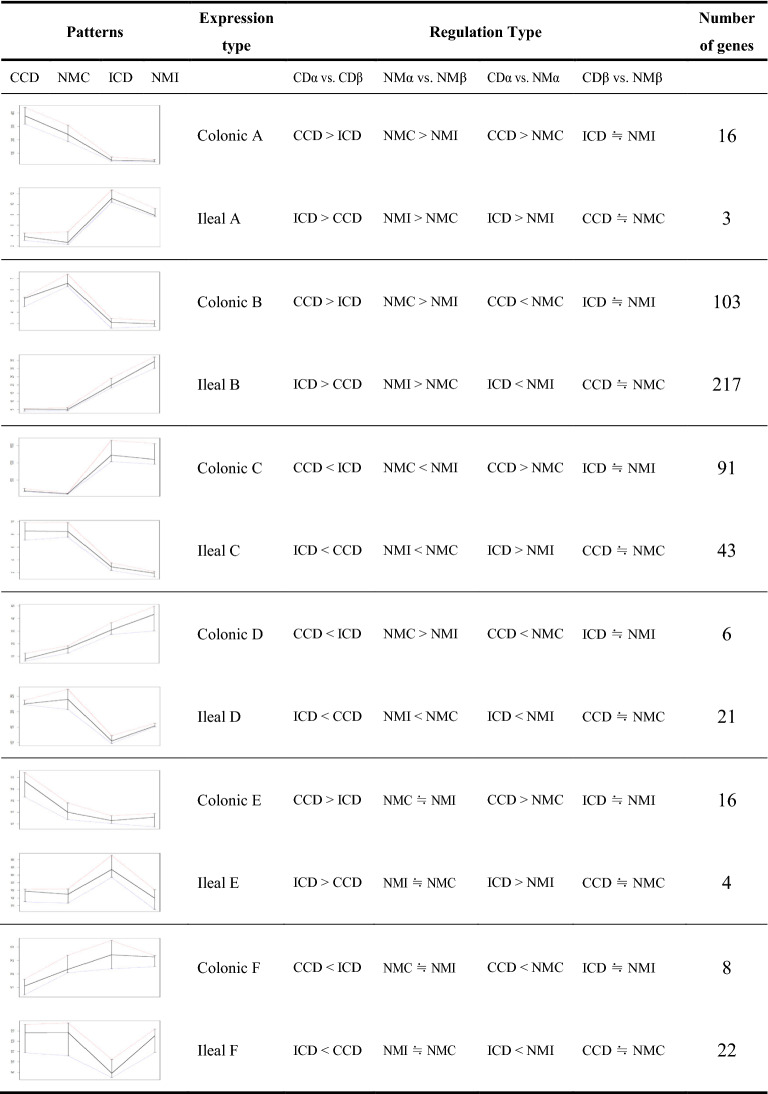
Summary of number of CCD and ICD-specific genes with different expression types

Depending on the tissue type of α and β, CCD and ICD-specific genes were separately classified: when α = colon tissue type and β = ileum tissue type, the result would be CCD genes; when α = ileum tissue type and β = colon tissue type, the result would be ICD genes. To get significantly differentially expressed genes, genes with *p*-value of 0.05 and confidence interval 95% were set to be cutoff for CDα vs CD*β*, NMα vs NM*β*, and CDα vs NMα. To specifically expressed, gene were also selected from CD_β_ vs. NM_β_, (*p*-value > 0.1). For each pattern graph, red, blue, and black line represents percentile of 75th, 25th, and median, respectively

CD: Crohn’s disease; NM: Normal (control) samples; CCD: colonic CD; NMC; normal colon; NMI: normal ileum; ICD: terminal-ileal CD; ≒: no differences (*p*-value > 0.1)

### Functional profiling of DEGs

**Fig. 5:**
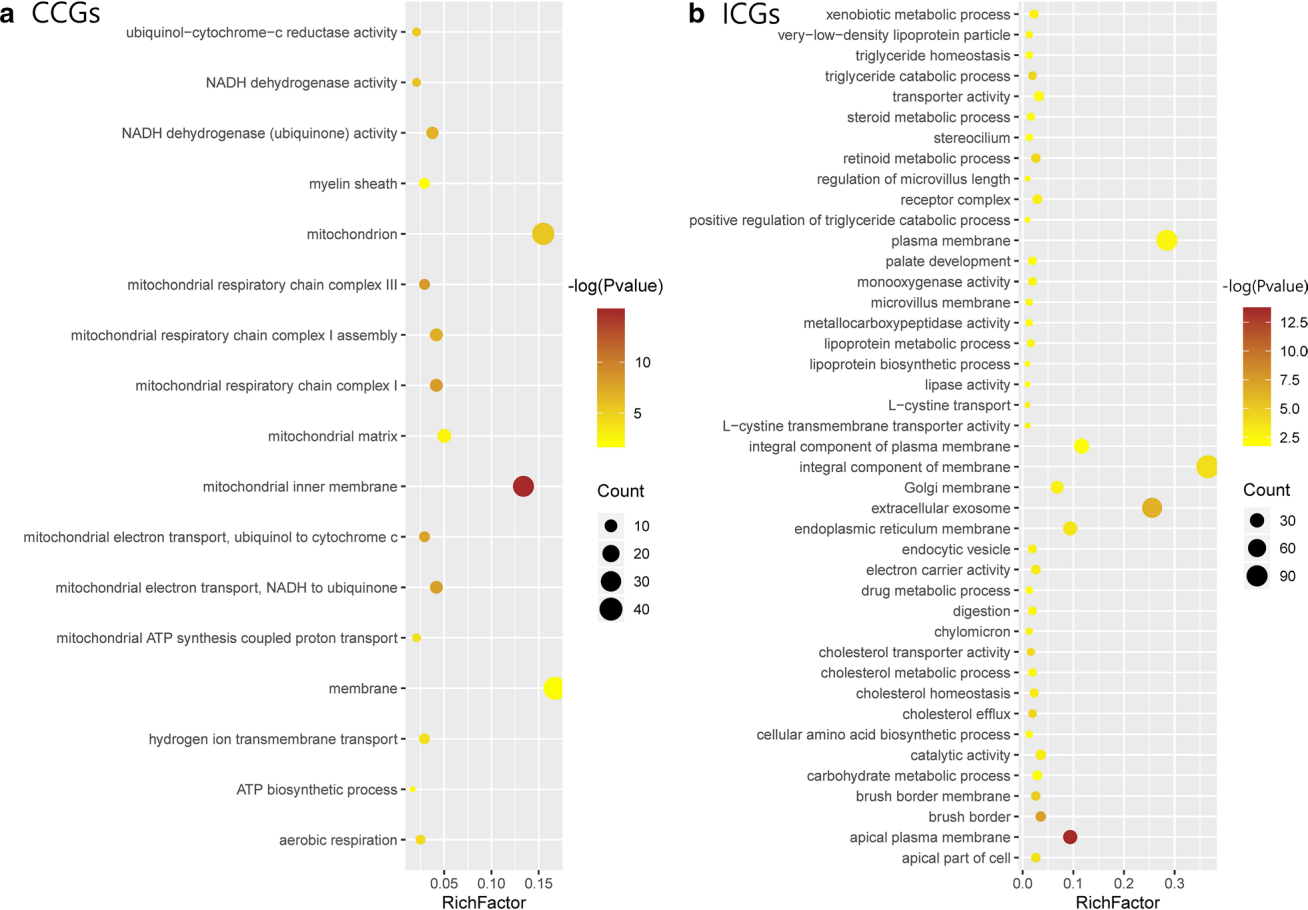
Gene ontology (GO) enrichment analysis of CCGs and ICGs. The x-axes represent the rich factor, which is the ratio of differentially expressed targeted gene numbers in the process to all the annotated genes located in the process. The higher rich factor indicates the higher level of enrichment. The dot size represents the number of target genes in the GO. **a** Scatter chart displaying Gene ontology of CCD genes, **b** Scatter chart displaying Gene ontology of ICD genes. *CCGs* colonic CD genes, *ICGs* terminal-ileal CD genes

**Fig. 6:**
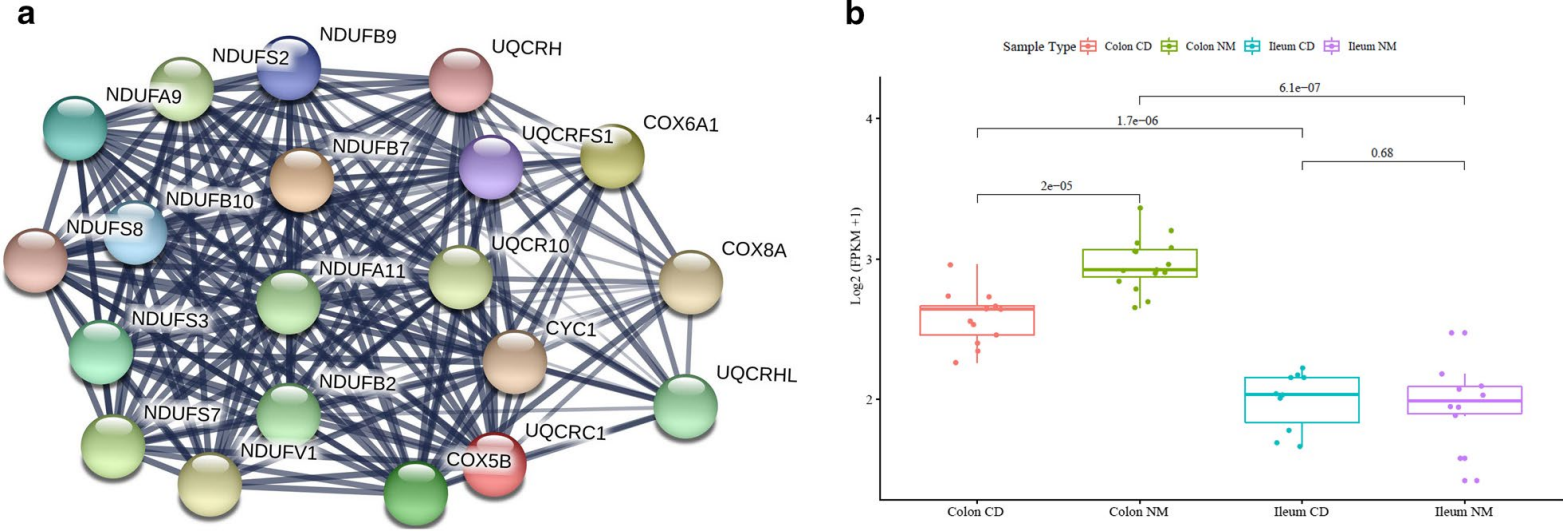
Protein–protein interactions among selected colonic CD genes (CCGs) involved in mitochondrial dysfunction. **a** The STRING graph shows 20 genes of mitochondrial dysfunction with interactions. Edges represent protein–protein associations and line width shows the strength of interaction. **b** Expression pattern of differentially expressed genes of type B. All 20 genes were belong to expression type B. *CCGs* colonic CD genes

As we verified differentially and specifically expressed genes in each tissue type that clearly defined each CCD and ICD, we hypothesized that there are also functional differences among DEG types. Therefore, we performed GO term enrichment analysis and KEGG pathway analysis to study disease location-related genes with having functional commonalities (n = 550). Using DAVID, we obtained 54 GO terms and 9 KEGG pathways for CCGs (Additional file 2: Table S5). Among the GO terms of the CCGs, three categories belonged to biological processes, four to cellular components, and three to molecular functions (false discovery rate ≤ 0.05). In the biological process category, mitochondrial functions were mainly among the top ten terms with respect to the number of DEGs in CCGs (Fig. 5a). According to KEGG pathway analysis, among nine pathways, the six most significant unexpectedly shared one functional commonality: mitochondrial dysfunction (Table 2). These pathways were related not only to reactive oxygen species levels and ATP production, but also to diseases such as NAFLD and neurological disorders including PD. In addition, 20 genes were involved in several mitochondrial complex deficiencies in both NAFLD and PD and were all downregulated, with type B: complexes I, III, IV, and V for PD and complexes I, III, and IV for NAFLD (Additional file 1: Fig. S8, Table 3) [19]. Mitochondrial complex deficiencies are a common feature of multiple diseases including myopathy, hypertrophic cardiomyopathy, liver disease, some forms of PD, NAFLD, and CD. We also repeated the analysis with ICGs to verify whether differences could be detected in GO terms and KEGG pathways compared to CCGs. Unlike CCGs, the GO term and KEGG pathway term only included ileum intestine function, such as digestion, absorption, and the metabolic process (Fig. 5b and Additional file 2: Table S6). Additionally, common genes in CD were mainly associated with immune and leukocyte function in biological process (Additional file 1: Fig. S9). As identifying biologically linked genes through GO analysis is complex, we supplemented genes from each CCD and ICD by STRING network analysis [20]. For CCGs, one major cluster was detected (Additional file 1: Fig. S10a), which included 20 of 240 CCGs and mitochondrial dysfunction (Fig. 6). Haberman et al. previously annotated 1696 downregulated genes in colon-only forms of IBD and revealed dysfunction in mitochondrial respiration [21]. Among the 220 CCGs, 60 genes overlapped and 13 genes of 20 genes related to mitochondrial dysfunction were detected.

**Table 2:** Most significant KEGG pathways with altered expression in terms of disease

Name of pathway	Count	*p*-value	Fold enrichment	FDR
CCGs				
Oxidative phosphorylation	22	3.18E−19	12.11	2.62E−14
^*^Parkinson’s disease	21	2.63E−17	10.82	1.81E−12
Alzheimer’s disease	22	4.60E−17	9.58	3.75E−12
*Non-alcoholic fatty liver disease (NAFLD)	21	1.12E−14	8.00	6.41E−10
Huntington’s disease	19	2.72E−14	9.21	1.05E−09
Metabolic pathways	41	7.24E−12	2.52	3.04E−06
ICGs				
Vitamin digestion and absorption	8	1.22E−7	18.53	1.52E−04
Fat digestion and absorption	41	3.57E−4	1.71	0.4450
Protein digestion and absorption	6	8.80E−4	7.84	1.0931
Metabolic pathways	8	1.63E−3	4.63	1.9248
Chemical carcinogenesis	7	4.53E−3	4.46	5.4888

^*^ Indicates the associations with CD were previously reported. Count: number of genes; *p*-value: modified Fisher Exact *p*-value, EASE Score, the smaller, the more enriched; Fold Enrichment: − log (*p*-value); CCGs: colonic CD genes; ICGs: terminal-ileal CD genes

**Table 3:** CD colon specific genes relates to mitochondria dysfunction (n = 20)

Gene symbol	Expression type	*p*-value		
		CCD vs. NMC	CCD vs. ICD	NMC vs. NMI
COX5B	B	1.17E−04	4.98E−04	5.45E−08
COX6A1	B	1.78E−04	5.37E−05	3.46E−07
COX8A	B	1.96E−04	1.44E−06	1.56E−08
CYC1	B	5.94E−06	4.76E−06	2.02E−10
NDUFA11	B	9.13E−04	2.12E−04	1.61E−07
NDUFA9	B	4.54E−04	6.79E−05	2.45E−06
NDUFB10	B	2.25E−04	7.07E−03	2.17E−06
NDUFB2	B	4.34E−04	2.80E−08	4.22E−08
NDUFB7	B	1.78E−04	5.24E−05	6.12E−07
NDUFB9	B	1.78E−04	5.75E−04	9.90E−07
NDUFS2	B	3.56E−05	3.10E−03	1.85E−07
NDUFS3	B	4.77E−04	7.60E−04	6.09E−06
NDUFS7	B	8.24E−04	1.39E−03	2.21E−07
NDUFS8	B	1.04E−03	3.11E−04	5.45E−07
NDUFV1	B	3.49E−08	9.42E−07	3.41E−13
UQCRC1	B	3.38E−04	8.44E−04	2.05E−06
UQCRH	B	1.19E−05	1.21E−03	7.42E−08
UQCRHL	B	2.30E−05	7.71E−07	8.57E−10
UQCR10	B	5.06E−04	6.70E−03	1.53E−06
UQCRFS1	B	3.33E−04	1.14E−03	1.29E−06

*CCD* colonic CD, *NMC* normal colon, *NMI* normal ileum, *ICD* terminal-ileal CD

### Excluding of UC characteristics

After characterizing CCD and ICD based on the DEGs, we used UC samples to filter UC characteristics based on DEGs to discriminate CD tissue-specific types. We analyzed UC samples vs. controls using the same approach of selecting CCD and ICD signatures (see “Materials and methods”), and identified 619 DEGs. We then compared UC DEGs with CCGs and ICGs. Most UC genes shared IBD characteristics (n = 274), and according to the segment, 209 genes were shared with CCGs and just 23 genes were shared with ICGs (Additional file 1: Fig. S11). Genes with IBD common characteristics were enriched in inflammatory response, integral component of membrane, and receptor activity (Additional file 2: Table S7-1). UC-specific genes were enriched in posttranscriptional regulation of gene expression, cell–cell junction, and copper ion binding (Additional file 2: Table S7-2). We filtered UC characteristics from the signature of CDs, and 318 genes remained including 31 CCGs and 287 ICGs (Additional file 1: Fig. S11). After eliminating UC characteristics from CD-specific genes, CCGs and ICGs with CD features remained, and they were prepared for discrimination.

### Discrimination of CCD and ICD

**Fig. 7:**
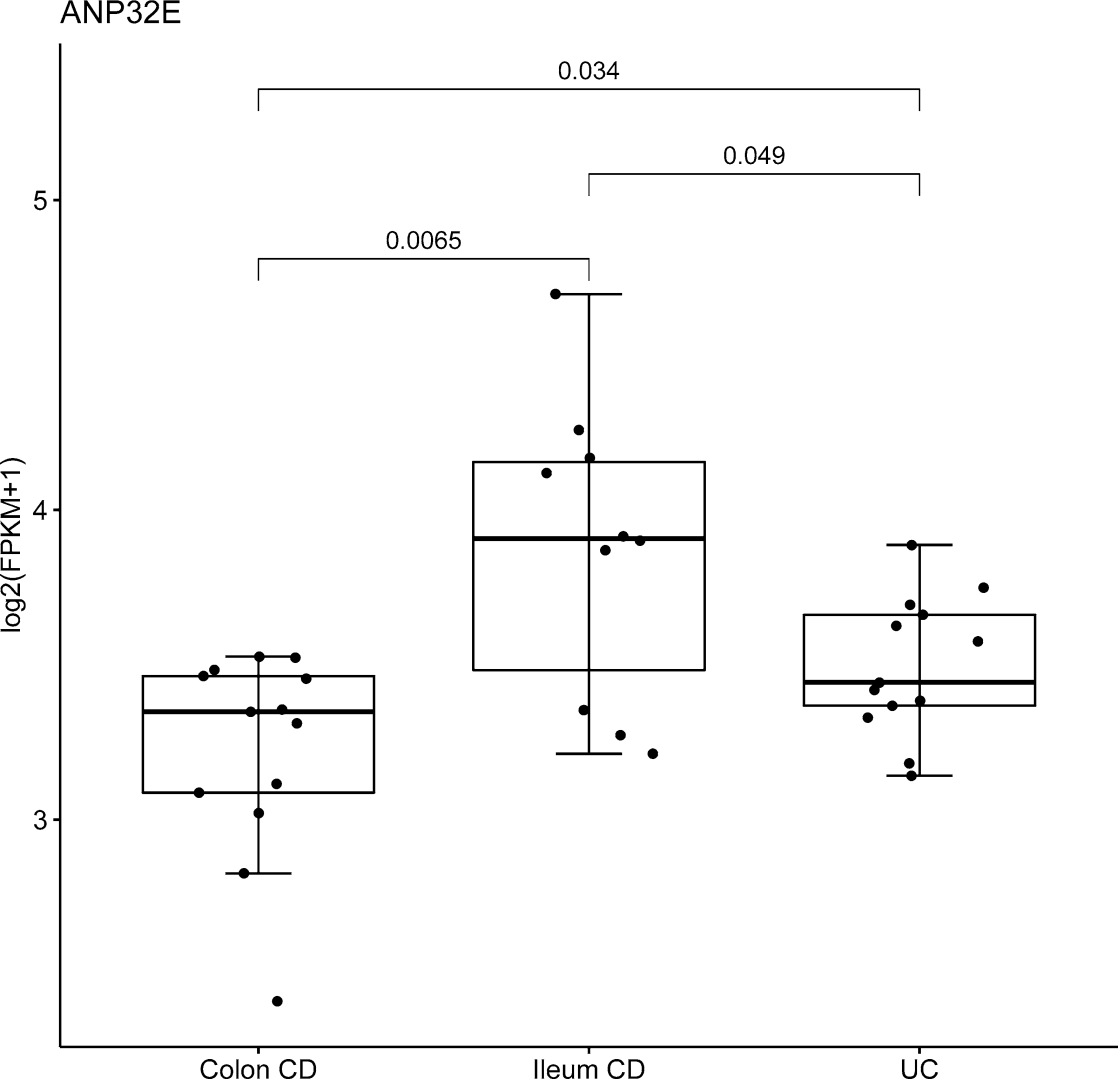
Final candidate genes that discriminate colonic CD from ileal CD and CD from UC. The boxplot of expression levels of ANP32E in the colon CD, Ileum CD, and UC samples of paediatric patients. Boxplots shows differences between three groups. Three group comparisons for each analysis are performed by Kruskal–Wallis test shown in *p*-values. *CD* Crohn’s disease, *UC* ulcerative colitis

Then we attempted to predict the CCD and ICD using these remained candidate genes using logistic regression (LR). All paediatric RNA-Seq data and candidate genes (n = 318) were used as input to calculate a list of genes suitable for CCD and ICD prediction. To derive and validate the model, we randomly divided the samples into a training set (70% of patients with CD and controls) and test set (remaining 30%). Because of the limited sample size, the training set was assessed by *K*-fold cross validation (*k* = 10). To choose the most effective gene, candidate genes were validated individually and selected by ten-fold cross validation, and then the selected genes were individually applied to the test set 100 times, confirming its strong prediction power as selected genes for discriminating CD subgroups (*p*-value ≤ 0.05, accuracy ≥ 0.8, and AUC ≥ 0.8). Thirty-three genes were selected for our final candidate genes (Additional file 2: Table S8). The results assured that each gene obtained by LR effectively differentiated the two molecular types in paediatric samples. Using these 33 genes, we predicted ICD and CCD by developing a discriminating model using random forest (RF) to calculate the performance of our final candidate gene sets, and the results also showed a strong prediction power (AUC = 0.833, accuracy = 0.857). We also evaluated the differences among CCD, ICD, and UC using the 33 final genes for confirmation based on the paediatric cohort (Fig. 7). Most genes showed significant differences between ICD and UC, as well as slight differences between CCD and UC. Therefore, the expression level of these selected genes could be useful when classifying CCD and ICD as well as distinguishing them from UC.

### Validation of the final candidate genes from other cohorts

To overcome the limited sample size, our study involved other paediatric cohorts to validate our final candidate genes. Similar to RNA-Seq data used in this study, we obtained data of the following two independent paediatric cohorts from the GEO database: GSE117993 with rectal biopsies (55 controls, 32 colonic CDs, 60 ileal-colonic CDs, and 43 UC samples) and GSE101794 with ileal biopsies (50 controls, 56 ileal CDs, 56 colonic CDs, and 142 ileal-colonic CDs) (Additional file 2: Tables S9 and S10-1). These patients with definite CD types were diagnosed as L1, L2, and L3 (i.e., L1 for the ileum, L2 for the colon, L3 for the ileocolon). In this study, we tried to characterize ileal-like CD (L1) and colon-like CD (L2) at the molecular level. Therefore, these data are relevant to confirm our results. According to the purpose of this study, we only acquired 56 ileal CDs (L1) from the ileal biopsy cohort and 32 colonic CDs (L2) from the rectal biopsy cohort. Ileocolon CDs (L3) were not included in our analysis, because these samples are represented in both ileal and colon segments.

Before analyzing the data by combining all cohorts, we tried to compare RNA-Seq data used in this study with those of other large cohorts to determine whether they are comparable enough to analyze together. We confirmed this using the sample clustering method. However, the independent cohorts showed differences (Additional file 1: Fig. S12). This may be due to the method of data processing, such as biopsy sampling and sequencing techniques. Therefore, we decided to use the same regression approach (*p*-value ≤ 0.05, accuracy ≥ 0.8, and AUC ≥ 0.8) on large cohorts to determine whether our final candidates are applicable in real-world. Consequently, among our final 33 candidate genes, 20 genes were identified in the large cohorts. These results suggested that the final 33 genes are sufficient to classify by gut segments. Moreover, we obtained high accuracy and AUC with the final 33 genes in the independent paediatric cohort data (0.93 and 0.92, respectively) (Table 4). This demonstrates that although our sample size was small, our results are comprehensive in other paediatric cohorts.

**Table 4:** Logistic regression analysis outcome from paediatric patients and adult patients

Patients	Number of genes	Accuracy	AUC	PPV	NPV
Paediatric	33	0.85	0.83	0.80	1
Paediatric validation	33	0.93	0.92	0.90	0.94
Adult validation	33	0.88	0.72	0.50	0.93

PPV = TP/TP + FP, NPV = TN/TN + FN

*AUC* area under the curve, *PPV* positive predictive value, *NPV* negative predictive valu**e**

Next, we evaluated if these selected genes were valid in data from adults. We used previously published colon and ileum microarray data of an independent cohort of adult patients with CD [22] to determine whether these final candidates could define CCD and ICD in an adult population (Additional file 2: Table S10-2). Unexpectedly, most genes still maintained a high accuracy and AUC (average 0.882 and 0.717, respectively; Table 4). Overall, our findings lay a foundation for discriminating CD tissue types in both paediatric and adult patients.

## Discussion

The discrimination between CD and UC has been widely examined but distinguishing CCD and ICD according to their phenotypes and location remains challenging in adults and children. Weiser et al. [18] improved the definition of CCD and ICD by describing colon-like CD and ileum-like CD. However, most previous studies of CD contains terminal ileum samples, as this is the most common localization [23]. For example, Haberman et al. [3] and Weiser et al. [18] identified 1281 ileal signature genes and 534 ileum-like genes, respectively. The aim of previous studies was to confirm the presence of CCD and ICD, not to characterize as well as discriminate their differences. In this study, we also observed two types of CD at the genetic level in paediatric RNA-Seq data through evaluation of our final candidates, functional profiling, and statistical analysis.

We investigated all possible protein-coding genes to select those that could discriminate the CCD and ICD by DEG analysis. The total numbers of CCGs and ICGs were 240 and 310, respectively. According to the expression atlas (https://www.ebi.ac.uk/gxa), 136 of 240 CCGs and 219 of 310 ICGs were identified previously, but the remaining genes require further analysis (Additional file 2: Tables S11 and S12).

We confirmed that there were both molecular and functional differences between the CCD and ICD and classified DEGs with six different patterns. Among these patterns, type B mostly consisted of both CCD and ICD, indicating that CCD and ICD-specific genes were all downregulated or inhibited. Moreover, the second most common type C showed opposite tissue features but these genes were upregulated with disease development. For instance, some genes functioning in the ileum function were upregulated in patients with CCD, whereas genes involved in colon function were upregulated in patients with ICD. Types E and F only responded to CD onset. Therefore, regardless of whether these genes play a role in intestinal functions under normal conditions, each represented features of its own tissue type. Studying these expression patterns can provide crucial evidence for determining the onset mechanism of each CCD and ICD.

IBD shows variable severity with relapses and remissions, leading to variable therapeutic decisions [24]. Each CCD and ICD also has a distinct therapeutic presentation, and paediatric patients with CCD are more likely to have severe symptoms, such as macroscopic inflammation and deep ulcers [18]. Through functional commonality analysis, we observed that all genes in the ICD were related to small intestine function. In contrast, genes from the CCD were associated with mitochondrial dysfunction, particularly in mitochondrial complex deficiencies, meaning that this tissue type can cause more severe symptoms by disturbing cellular metabolic homeostasis [25]. Mitochondrial dysfunction exacerbates inflammation and barrier dysfunction though inflammatory stimuli by affecting mitochondrial metabolic functions [26, 27]. The mitochondrial complex converts chemical energy from food into ATP through oxidative phosphorylation. Deficiencies in mitochondrial complexes such as complex I, III, IV, and V may cause various problems in the brain, liver, and muscles and have been associated with encephalomyopathy and hydrocephalus [28–31]. Previous studies showed that not only the progression of colonic dysplasia in UC accompanied mitochondrial loss [32] but also the mitochondrial gene expression was downregulated only in colonic IBD, including CD and UC rather than ICD or both ileal and colonic inflammation [21], supporting our results showing suppression of only genes related mitochondrial complexes in CCD. Moreover, inflammatory mediators interrupt mitochondrial metabolism and impair mitochondrial function which increase the inflammatory response, resulting in neurodegenerative disorders [33]. According to our functional analysis, PD and NAFLD share mitochondrial deficiencies in complexes I, II, and IV, and PD presents a complex V deficiency. Complex V deficiency is known to cause muscle pain [34]. In support of our results, recent research showed that neurodegenerative disorders may start in a highly localized segment of the GI tract, where the alpha-synuclein aggregation/degradation balance is shifted by the presence of inflammation [35]. Although Alzheimer’s disease and Huntington’s disease also showed an association with the CCD, their associations with CD have not been reported; they have only been studied in terms of mitochondrial dysfunction. Therefore, additional functional studies of these 19 genes involved in mitochondrial complex deficiencies could contribute to an understanding of the pathophysiology of brain, liver, and muscle-related diseases. Additionally, the results of functional profiling of CD common genes revealed that two different CD tissue types are involved in the immune response but with different functions. Further studies of these genes are needed to explore their association with the two different tissue types. Based on our results, the CCD and ICD were molecularly and functionally different, enabling selection of final candidates for characterizing the two types.

Along with clinical features, some CCD showed inflammatory disease limited to the mucosa, without mural involvement, reminiscent of UC, named as ‘UC-like Crohn’s disease’. Particularly, these patients were significantly younger than those with mural involvement [36]. Therefore, some candidate genes of CCGs could distinguish not only between CCD and ICD but also between CCD and UC. To distinguish CCD and ICD from other IBDs, we used UC samples to filter UC characteristics from DEGs. By eliminating UC characteristics, 318 genes remained after filtration, including 33 CCGs and 287 ICGs. From the results of filtration, it was confirmed that most CCGs have marked similarity in CCD and UC, as expected. Recently, Matthew and his colleagues classified CD into two clinically relevant subtypes [18]. In their study, they identified 849 (315 for colon and 534 for ileum) DEGs between colon-like and ileum-like CD using transcriptome data. By comparing our results with previously annotated CCD and ICD, we found that 116 genes were overlapped for ICD, but only two genes overlapped in CCD. From these results, it seems that among the results of previous study, many genes similar to the characteristics of UC were included. Therefore, the final candidate genes of this study could be utilized as markers to understand the diverse phenotypes of CD independently of UC.

Despite the limited number of paediatric samples, two distinct molecular phenotypes were found. In addition, the filtration of UC features strengthens the discrimination of CD from other IBDs. By evaluating differentially and specifically expressed genes, we built a discriminatory model for CCD and ICD using LR. Consequently, 33 genes were obtained with an AUC and accuracy of 0.83 and 0.86, respectively. These results confirmed that each gene identified from LR effectively differentiated the CCD and ICD in paediatric samples. To determine whether the classification power of the selected genes could be extended to other cohorts, our study involved data of rectal and ileal biopsy samples from an independent cohort of paediatric patients, and microarray data from adult colon and ileum samples. The performance of the 33 genes in another paediatric cohort was high (AUC and accuracy of 0.92 and 0.93, respectively). As the sample size increases, the overall accuracy seems to increase. This is because the smaller the number of samples, the greater the effect of one false prediction value on accuracy. Moreover, the results of independent paediatric cohort demonstrate the 33 genes have the power to characterize CCD and ICD in paediatric patients with CD. From the adult microarray data, a reliable performance of discrimination between CCD and ICD was also obtained although the age and clinical effects in both populations were different. This strongly suggests that because the characteristics of treatment-naïve patients with CD were conserved among heterogeneous adult patients and under various conditions, such as treatments and other factors.

In addition, most of final candidate genes were also previously reported to be related to CD according to tissue types (Additional file 2: Table S13). ERAP1, one of the final candidate CCGs, was recognized for its role in innate immune-mediated pathways involved in inflammatory responses [37]. Some ICGs, such as *BDH2, CYP4V2, OIT3, PLD1* and *SLC25A23*, were reported as differentially expressed in ileum tissue from Crohn’s disease vs. non-inflammatory bowel disease control [38–42]. Furthermore, some genes were identified for their association with CD or IBD according to the dataset of atlas-experiments (https://www.ebi.ac.uk/gxa).

Although the number of patients was restricted in this study, reliable discrimination between the CCD and ICD was obtained based on another independent adult cohort. This is the first study to characterize each CD location type at the genetic level by DEGs based on six different expression patterns, functional differences, and classification power for both paediatric and adult cohorts. Overall, our findings improve the understanding of the diverse phenotypes of CD independently of UC.

The objective of this study was to identify gastrointestinal tract-specific gene expression signatures characterized for CCD and ICD types using transcriptome data (RNA-Seq). In summary, we identified the DEGs that are specifically expressed in CCD and ICD compared to those in healthy controls. By defining gene expression profiles of general UC, we could discriminate CCD- and ICD-specifically expressed genes. In addition, a classification model was built using LR to select the most effective gene set and classify CCD and ICD. Consequently, 33 genes were obtained as useful for discriminating CCD and ICD with high accuracy and AUC values not only for paediatric patients but also for an independent cohort of adult patients. We propose that our approach and the final gene set identified for the two CD locations are useful for the classification of CD patients. Furthermore, our findings improve the understanding of the diverse phenotypes of CD independently of UC. A further study with larger sample sizes will provide a better understanding of the cellular and molecular mechanisms involved in the regulation of CCD and ICD and might be crucial for personalized treatment of CD.

## Materials and methods

### Gene expression datasets

Crohn's disease RNA-Seq data were derived from colons and terminal ileums of children newly diagnosed with CD and children without IBD with full ethical approval in the University of Cambridge Department of Paediatrics (Registration February 2018) and downloaded from the NCBI Sequence Read Archive (BioProject: PRJEB24645). The appropriate ethics review board approved all study participants provided informed consent and all methods were performed in accordance with the relevant guidelines and regulations. In total 79 samples, 25, 27, and 27 samples were CD, controls, and UC, respectively. Among them, we selected only 22 samples with CD, 13 samples with UC and 27 healthy control samples: colon tissues from 15 CD samples, 13 UC samples and 16 controls, and terminal ileum tissues from 10 CD samples and 11 controls. All terminal ileum biopsies were extracted from terminal ileum, and colon biopsies contain ascending and sigmoid region. Analysis was performed using integrated colonic samples. Detailed information about the patients, such as age, individuals, sex, and region, is shown in Additional file 2: Table S1. Downloaded SRA files were converted to FASTQ files using the SRA toolkit (version 2.8.2) available from https://github.com/ncbi/sra-tools. To validate final candidates, we used 32 CD samples and (GSM3316656–GSM3316687) 43 UC samples (GSM3316803–GSM3316845) of paediatric patients rectal biopsies from the Gene Expression Omnibus (GEO) database (GEO accession: GSE117993) and 56 CD samples of paediatric patients ileal biopsies from GEO database (GEO accession: GSE101794) (Additional file 2: Table S10). We also downloaded microarray data from 8 colon samples (from GSM1426079–GSM1426089) 51 active ileum samples (from GSM1945759–GSM1945809) of adult patients with CD from GEO database (GEO accession: GSE75214) and the study was approved by the ethics committee of the UZ/KU Leuven.

### Data analysis

Cutadapt v1.15 was used to trim the adapter sequence from the sample data (minimum length = 25 bp, Phred score > 20). FastQC v0.11.8 (www.bioinformatics.babraham.ac.uk/projects/fastqc/) was used to check the sequence characteristics and quality distribution [43]. Trimmed RNA-Seq reads were then aligned to human genome assembly GRCH38p.11 (www.ncbi.nlm.nih.gov/grc/human/data/) using HISAT2 v2.1.0 [44]. On average, RNA-Seq analyses produced 17,292,896 reads per sample, of which 93.45% were on target and mapped to the reference genome. Mapping statistics were compared across all disease and control samples. Obtained transcripts were quantified using Cuffquant and Cuffnorm with default parameters to calculate expression values and for normalization (version 2.2.1). Protein-coding genes were selected from these data according to the Ensembl database (https://ensembl.org/Homo_sapiens/). We calculated the sum of mean fragments per kilobase million (FPKM) across all samples for each protein-coding gene. If the maximum values of this sum across all samples were below 1, the gene was discarded. Two CCD samples of outliers were excluded according to an initial PCA and correlation plot. For multiple testing problems, four types of DEGs in CD samples relative to control in each tissue locations were identified by the Levene’s test and Shapiro test with *t* test and Wilcoxon signed-rank test in Bioconductor R (www.bioconductor.org/). Statistical analysis of DEGs used a *p*-value threshold of 0.05. In addition, to obtain a precise range of true values, 95% CIs were calculated for each case and genes were selected when the lowest CI value (LCI) of one sample type was larger than the highest CI value (HCI) of the other sample type in each case. This process was performed with all colon samples considering all ileum samples first, to filter false-positive genes, and then repeated on all four different pairwise comparisons.

### Functional profiling analysis

To analyze the functions of these DEGs and their involvement in various biological parameters (molecular functions, biological processes, or cellular components), the DEGs were classified into categories according to the Gene Ontology (GO) database [45]. Moreover, the functional mechanisms of the DEGs were investigated by Kyoto Encyclopedia of Genes and Genomes (KEGG) pathway [46] analysis and the Database for Annotation, Visualization and Integrated Discovery DAVID web server [47]. Fisher’s exact *t*-test was used for enrichment analysis. Finally, the protein–protein interaction network encoded by DEGs was obtained using the Search Tool for the Retrieval of Interacting Genes/Proteins (STRING, http://string-db.org/) [20].

### Classifying expression patterns

To characterize genes specifically expressed in each disease location, several expression patterns were classified along with tissue features. Six expression patterns were categorized for each tissue type and applied to the dataset for obtaining a heatmap with log_2_ normalization. Unsupervised hierarchical cluster methods using a Pearson distance and Spearman’s correlation were applied for gene and sample classification, respectively, to visualize and compare each gene expression pattern across different sample types.

### UC filtration

UC samples were used to filter UC features from DEGs. Statistical tests, such as a t-test and Wilcoxon signed-ranked test, were applied to the analysis with a *p*-value threshold of 0.05.

### Statistical methods

Comparative analysis between CCD and ICD was performed using LR. The CCD and ICD specific genes identified by paediatric data analysis were used to differentiate between CD colon and ileum types. The dataset was divided so that 70% of the data were used to train the model and 30% to evaluate its performance. *k*-Fold validation on the training set was used to achieve explicit results as well as to complement the limited sample sizes (*k* = 10). A dataset with n samples in the training set was split in *k* equal-sized partitions. The number of training samples for each partition is n/k, and they must appear in the validation set only once. *p*-values ≤ 0.05 were considered as statistically significant. The accuracy and AUC of each gene were analyzed in test set, and 0.8 was set as the minimal cut-off value. This process was repeated 100 times for the training set to select the final genes and evaluate the performance of the test. Moreover, to validate the results, random forest (RF) was applied to another paediatric RNA-Seq data and adult microarray data as independent cohorts. The accuracy and AUC of these analyses were also calculated to determine the classification power of our final candidate genes.

## Supplementary Information

**Additional file 1.** Additional Figures S1–S12.

**Additional file 2.** Additional Tables S1–S13.

## Data Availability

The datasets supporting the conclusions of this article are included within the article and its additional files.

## Abbreviations

CD: Crohn’s disease
UC: Ulcerative colitis
IBD: Inflammatory bowel disease
LR: Logistic regression
CI: Confidence interval
AUC: Area under curve
NAFLD: Non-alcoholic fatty liver disease
DEGs: Differentially expressed gene
LCI: The lowest CI value
HCI: The highest CI value
PCA: Principal component analysis
CCD: Colonic CD
ICD: Terminal-ileal CD
NMC: Colon control
NMI: Ileal control
CCGs: CCD genes
ICGs: ICD genes
