# Correction to: Characterization of terminal-ileal and colonic Crohn’s disease in treatment-naïve paediatric patients based on transcriptomic profile using logistic regression

**DOI:** 10.1186/s12967-021-02945-9

**Published:** 2021-08-03

**Authors:** Ilkyu Park, Jaeeun Jung, Sugi Lee, Kunhyang Park, Jea-Woon Ryu, Mi-Young Son, Hyun-Soo Cho, Dae-Soo Kim

**Affiliations:** 1 Department of Bioinformatics, KRIBB School of Bioscience, Korea University of Science and Technology (UST), 217 Gajeong-ro, Yuseong-gu, Daejeon, Korea; 2 Department of Environmental Disease Research Center, Korea Research Institute of Bioscience and Biotechnology (KRIBB), 125 Gwahak-ro, Yuseong-gu, Daejeon, 34141 Korea; 3 Department of Core Facility Manage-ment Center, Korea Research Institute of Bioscience and Biotechnology (KRIBB), 125 Gwahak-ro, Yuseong-gu, Daejeon, Korea; 4 Department of Rare Disease Research Center, Korea Research Institute of Bioscience and Biotechnology (KRIBB), 125 Gwahak-ro, Yuseong-gu, Daejeon, Korea; 5 Department of Stem Cell Convergence Research Center, Korea Research Institute of Bioscience and Biotechnology (KRIBB), 125 Gwahak-ro, Yuseong-gu, Daejeon, Korea

## Correction to: J Transl Med (2021) 19: 250 https://doi.org/10.1186/s12967-021-02909-z

In the original publication [1] the corresponding authorship of Mi-Young Son was accidentally omitted during the publication process.

The original article has been updated. We apologize to the authors and readers for the inconvenience.
