# Supplementary material for: Characterization of terminal-ileal and colonic Crohn’s disease in treatment-naïve paediatric patients based on transcriptomic profile using logistic regression
Source: J Transl Med. 2021 Jun 7;19:250. doi: 10.1186/s12967-021-02909-z (PMC8185924; doi:10.1186/s12967-021-02909-z)
Supplement: Supplementary file 1 — Additional file 1. Additional Figures S1–S12. [file 12967_2021_2909_MOESM1_ESM.pdf]

Supplementary Tables:

Supplementary Table 1: RNA-seq mapping statistics for each individual patient sample.

Supplementary Table 2-1: Summary of numbers of differentially expressed genes of CD vs. Controls in colon and ileum samples, respectively.

Supplementary Table 2-2: Summary of numbers of differentially expressed genes of Colon vs. Ileum in CD and Controls, respectively.

Supplementary Table 3: Differentially expressed genes with expression Type of Colonic CD genes.

Supplementary Table 4: Differentially expressed genes with expression Type of Terminal-ileal CD genes.

Supplementary Table 5: Gene Ontology and KEGG pathways for CCGs.

Supplementary Table 6: Gene Ontology and KEGG pathways for ICGs.

Supplementary Table 7-1: The independent validation cohort from the Gene Expression Omnibus database (GEO accession: GSE117993 and GSE101794)

Supplementary Table 7-2: The independent validation cohort from the Gene Expression Omnibus database (GEO accession: GSE75214)

Supplementary Table 8: Performance statistic from machine learning methods for 33 genes in paediatric cohort.

Supplementary Table 9: Summary of CD transcriptomic datasets and their use for validation of the current study results.

Supplementary Table 10-1: The independent validation cohort from the Gene Expression Omnibus database (GEO accession: GSE117993 and GSE101794).

Supplementary Table 10-2: The independent validation cohort from the Gene Expression Omnibus database (GEO accession: GSE75214)

Supplementary Table 11: Expression atlas results of CCGs.

Supplementary Table 12: Expression atlas results of ICGs.

Supplementary Table 13: Final candidate genes and their description and studies

Supplementary Figures:

Supplementary Figure 1: Correlation plot from Colonic CD (a) and Terminal-leal CD (b)

Supplementary Figure 2: Principal component analysis of each tissue type **Supplementary**

Figure 3: Density plot from Colonic CD vs. Terminal-ileal CD

Supplementary Figure 4: Venn diagram with four different comparisons

Supplementary Figure 5: Venn diagram of ileal CD genes with DEGs from two other studies, Haberman et al., and Weiser et al.

Supplementary Figure 6: A heatmap of both CCSGs and CISGs in four different sample types

Supplementary Figure 7: Boxplots of six different expression types in colonic CD genes (CCGs) and termianl-ileal CD genes (ICGs)

Supplementary Figure 8: Two major disease of KEGG Pathway from CCGs

Supplementary Figure 9: Bar chart displaying biological function category from Gene ontology analysis of common genes (n=471) for Colonic CD and Terminal-Ileal CD

Supplementary Figure 10: Protein-protein interaction network for CCGs (n=240) and ICGs (n=310)

Supplementary Figure 11: A venn diagram illustraing differentially expressed genes in colonic CD, ileal CD , and UC

Supplementary Figure 12: : Dendrogram of CD patients from different cohorts.

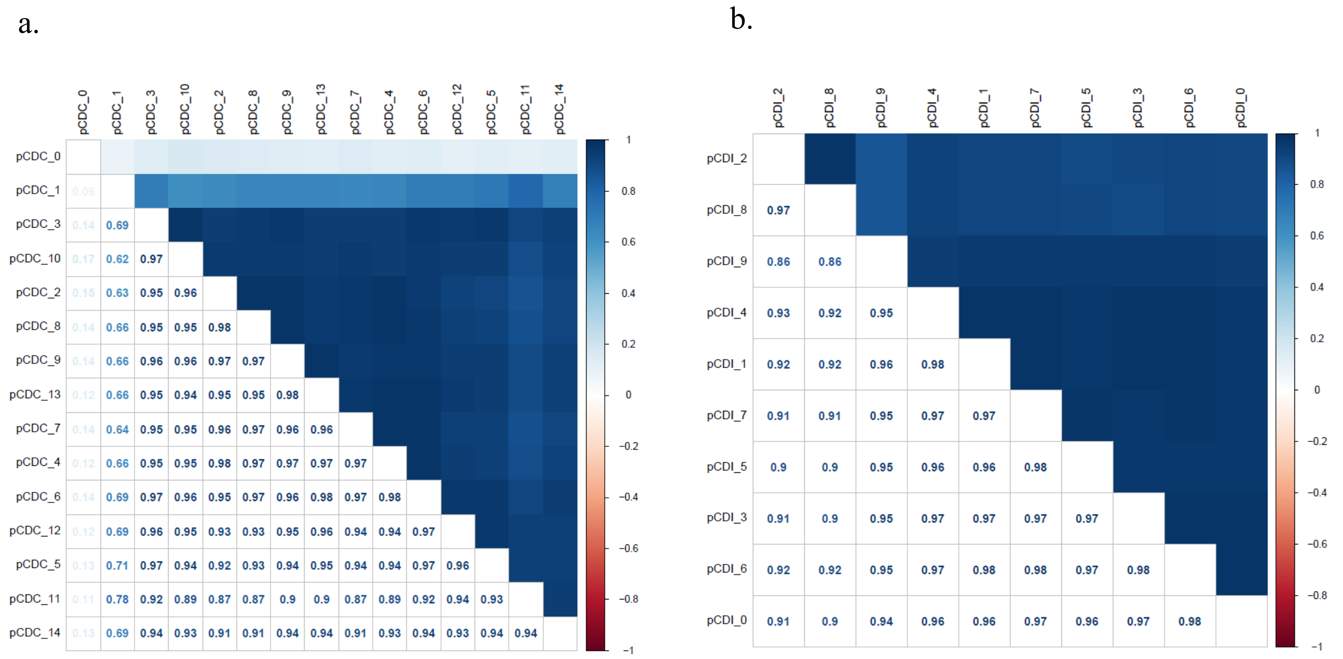

Fig. S1. **Correlation plot from Colonic CD (a) and Terminal-leal CD (b)**

The correlation plot from colonic CD samples(a) and terminal-ileal CD samples (b) were shown above. (a) Among 15 total colonc CD samples, two samples had lower than average of 0.7 compared to other samples. Therefore, these two samples were discarded on the analysis. (b) Total 10 samples were used to display correlation plot and no samples were removed.

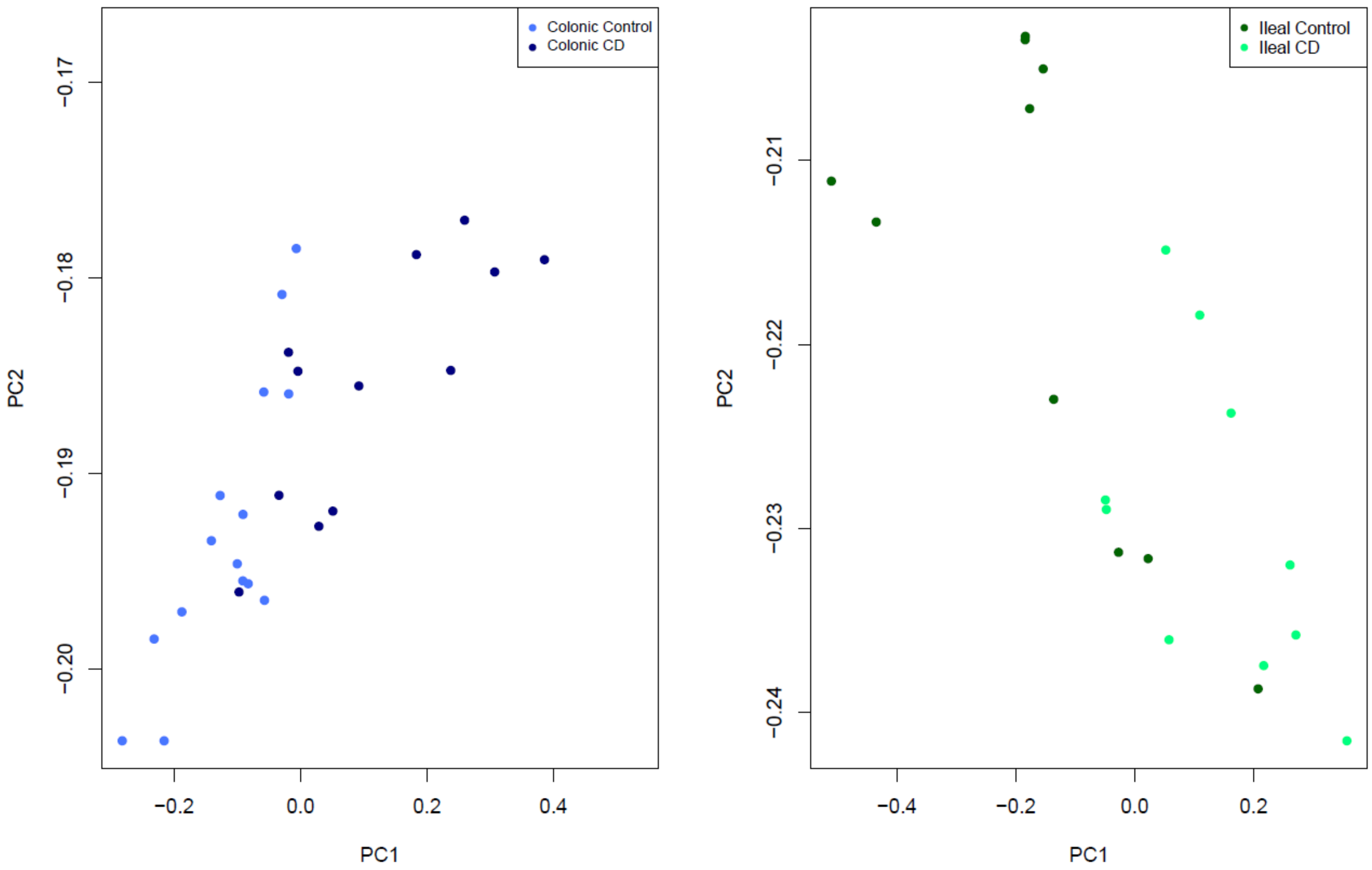

Fig. S2. Principal component analysis of each tissue type

Unsupervised principal component analysis analysis showing difference between colonic control (royal blue) and colonic CD (navy blue), and terminal-ileal control (spring green) as well as and terminal-ileal CD (dark green).

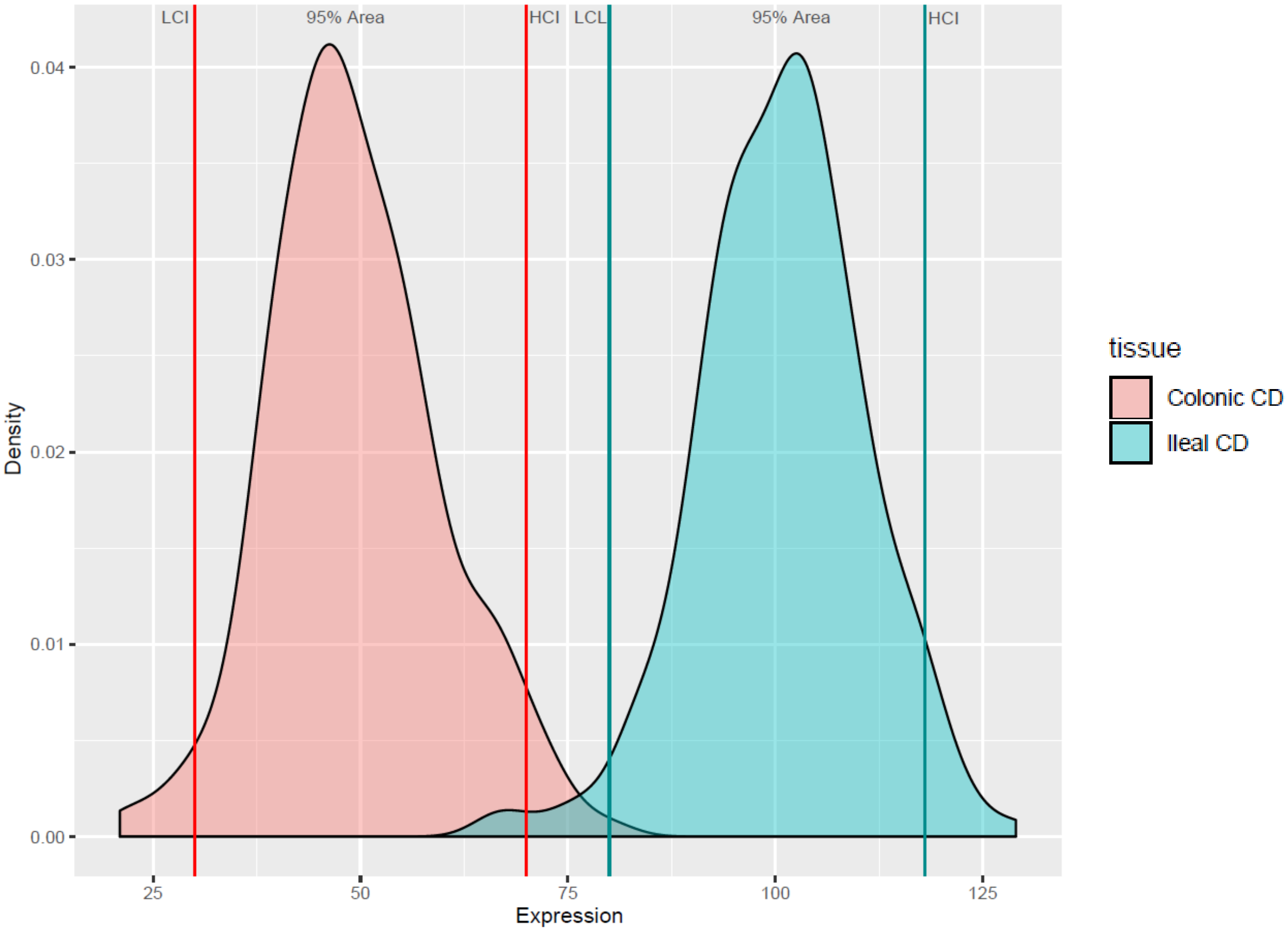

Fig. S3. **Density plot from Colonic CD vs. Terminal-ileal CD**

This density plot was an example of how gene were selected. Lowest confidence interval (LCI) and highest confidence interval (HCI) were calculated; 30 – 70 and 80 – 118 were computed for colonic CD and terminal- ileal, respectively. Genes were selected when their CI 95% area didn't overlap one another.

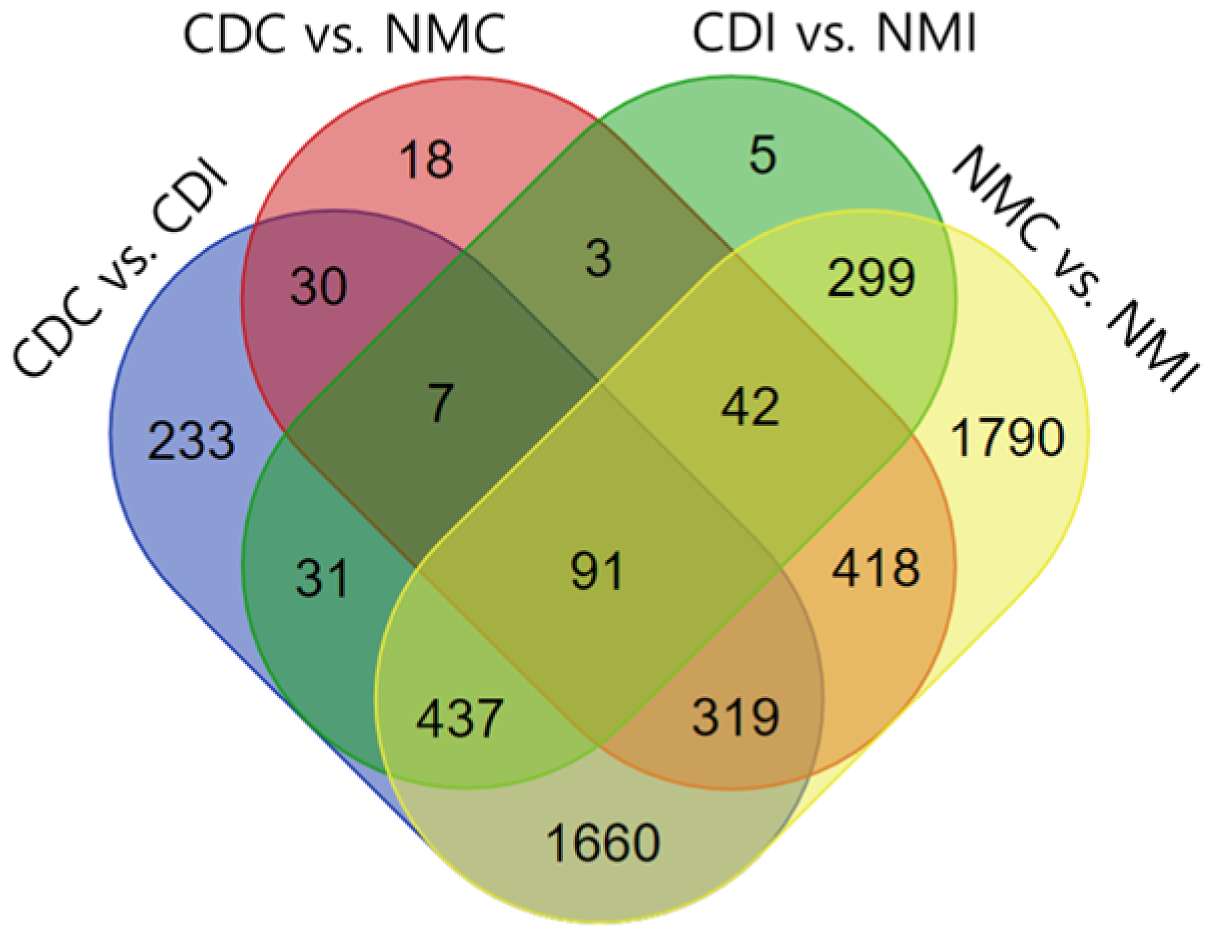

Fig. S4. **Venn diagram with four different comparisons**

Four pairwise comparison were compared in venn diagram; colonic CD (CDI) versus. ileal CD (CDI) (blue); colonic CD (CDI) versus. colon control (NMC) (red), ileal CD (CDI) versus ileal control (NMI) (green), colon control (NMC) versus ileal control (NMI) (yellow). The genes differentially expressed between CDC and NMC and between CDI and NMI were selected among the DEGs between CDC and CDI. There are six cases which could represent differences between CDC and CDI, intersection of all comparisons (91 genes), intersection of CDC vs. CDI and CDC vs. NMC (30 genes), intersection of CDC vs. CDI and CDC vs. NMC and CDI vs. NMI (7 genes), intersection of CDC vs. CDI and CDI vs. NMI (31 genes), intersection of CDC vs. CDI and CDI vs. NMI and NMC vs. NMI (437 genes) and intersection of CDC vs. CDI and CDC vs. NMC and NMC vs. NMI (319 genes).

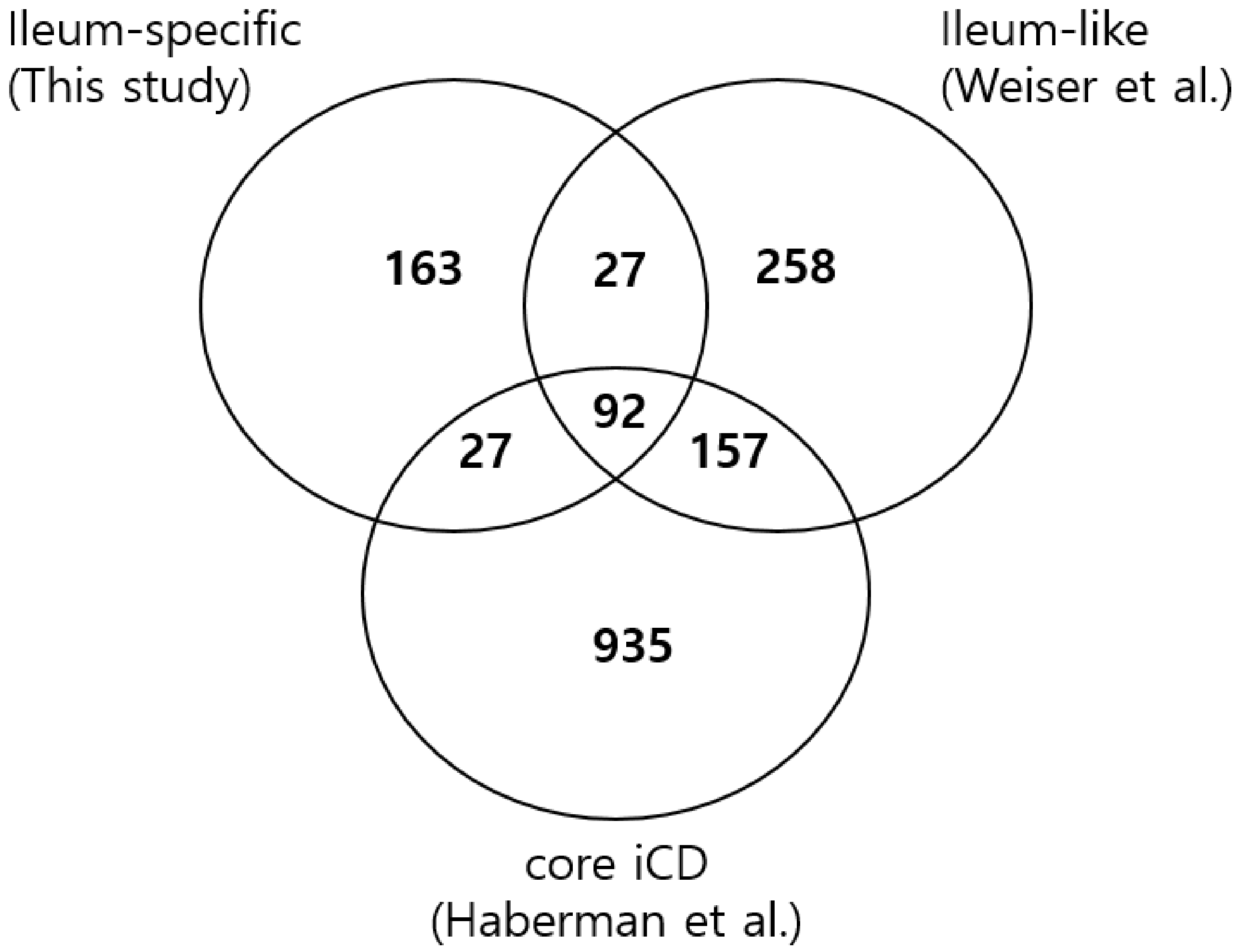

Fig. S5. Venn diagram of ileal CD genes with DEGs from two other studies, Haberman et al., and Weiser et al.

Haberman et al.(core iCD) and Weiser et al.(ileum-like) identified 1,281 ileal signature genes and 534 ileum-like genes, respectively. Our results of ileum CD specific genes (310) were compared with ileal CD gene from previous found known, and 119 genes were overlapped with each study and a total of 92 genes were observed in all studies containing our result.

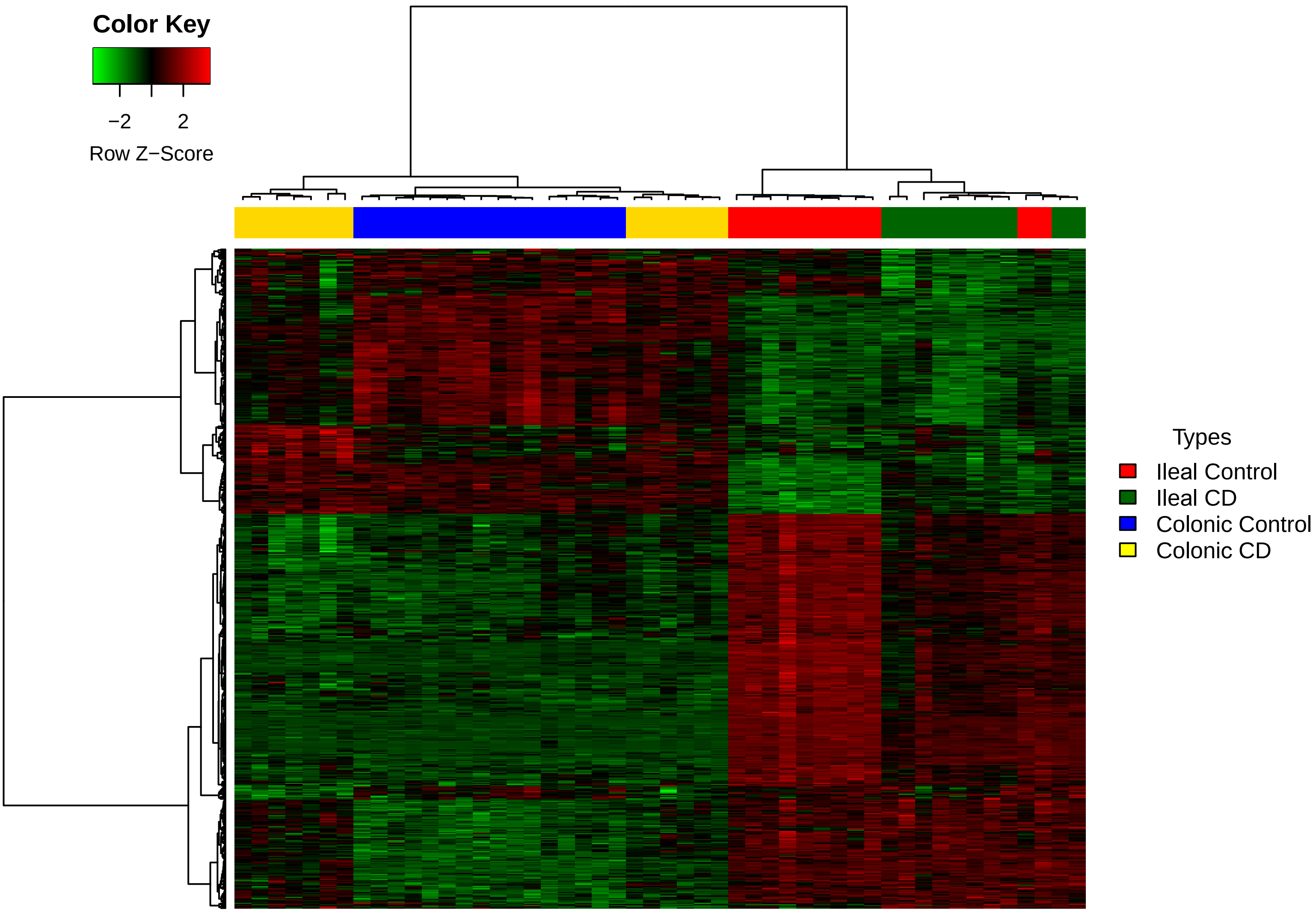

Fig. S6. A heatmap of both CCSGs and CISGs in four different sample types

A heatmap of both CCSGs and CISGs across controls and CD patient subgroup. Samples are color-coded in the top bar according to the sample types and diagnosis.

a.

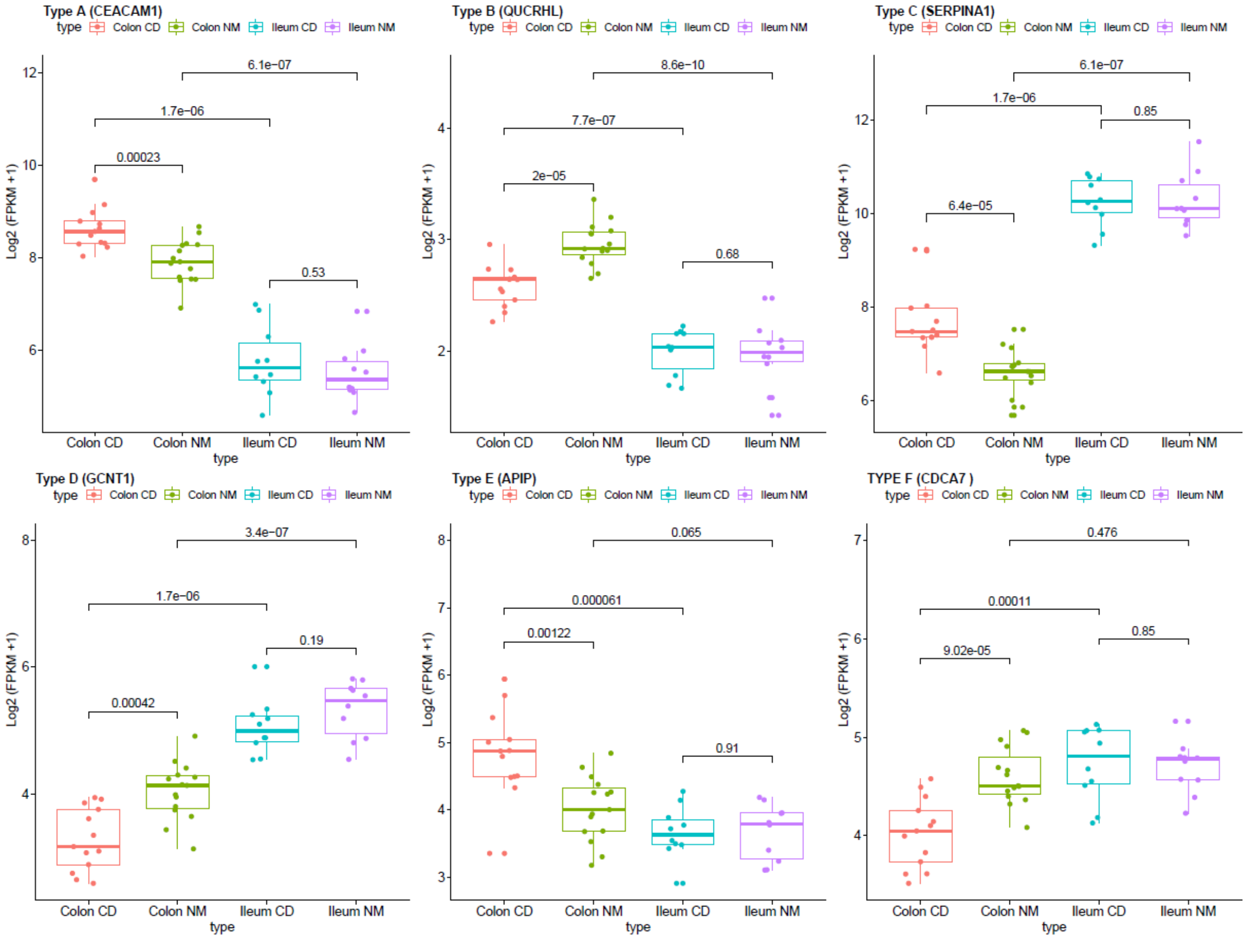
Type A (CEACAM1)
type Colon CD Colon NM Ileum CD Ileum NM
Log2 (FPKM +1)
6.1e−07
1.7e−06
0.00023
0.53
Colon CD Colon NM Ileum CD Ileum NM
type
Type B (QUCRHL)
8.6e−10
7.7e−07
2e−05
0.68
Type C (SERPINA1)
6.1e−07
1.7e−06
0.85
6.4e−05
Type D (GCNT1)
3.4e−07
1.7e−06
0.19
0.00042
Type E (APIP)
0.065
0.000061
0.00122
0.91
TYPE F (CDCA7)
0.476
0.00011
0.85
9.02e−05

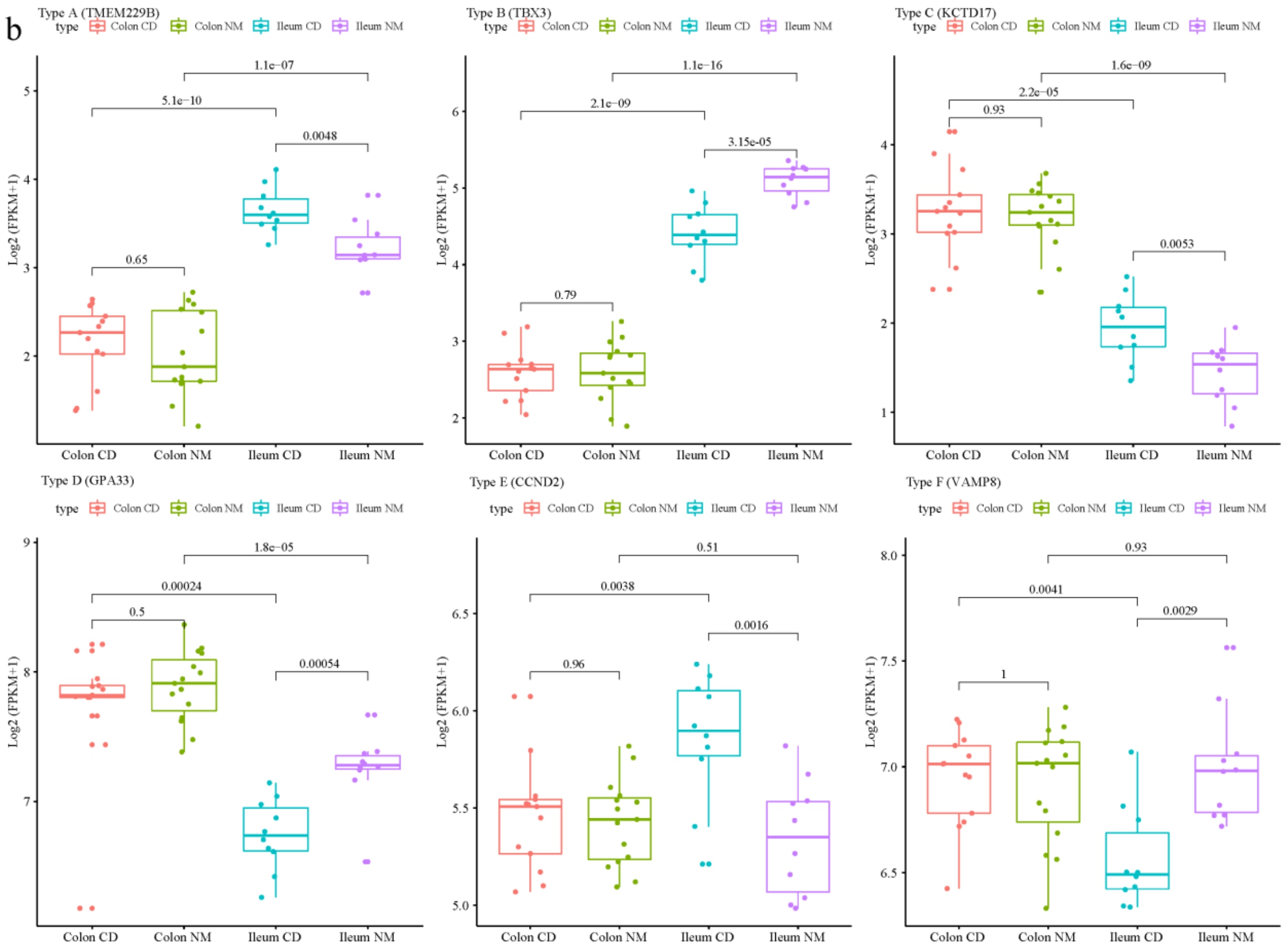

Fig. S7. Boxplots of six different expression types in colonic CD genes (CCGs) and termianl-ileal CD genes (ICGs)

(a) Boxplots of colonic CD Genes. Gene were selected to exhibit six different expression types according to the most significant differences for all three cases: colon CD vs. colon NM, colon CD vs. Ileum CD, and colon control vs. ileum control. Type A and B were up-regulated in both colon CD and colon controls compared to ileum samples. Type A was up-regulated in colon CD compared to colon controls (n = 15). Type B was down-regulated in colon CD compared to colon controls (n = 103). Type C and D were down-regulated in both colon CD and colon controls compared to ileum samples. Type C was up-regulated in colon CD compared to colon controls (n= 91). Type D was down-regulated in colon CD compared to colon controls (n= 6). Type E and F were that there were no differences among colon controls, ileum controls, and ileum CD samples. Type E was colon CD up-regulated genes (n= 16). Type F was colon CD down-regulated genes (n=8). **(b)** Boxplots of Ileal CD genes. Gene were selected to exhibit six different expression types according to the most significant differences for all three cases: ileum CD vs. ileum NM, ileum CD vs. colon CD, and ileum control vs. colon control. Type A and B were up-regulated in both ileum CD and ileum controls compared to colon samples. Type A was up-regulated in ileum CD compared to ileum controls (n = 3). Type B was down-regulated in colon CD compared to colon controls (n = 217). Type C and D were down-regulated in both ileum CD and ileum controls compared to colon samples. Type C was up-regulated in ileum CD compared to ileum controls (n = 43). Type D was down-regulated in ileum CD compared to ileum controls (n = 34). Type E and F were that there were no differences among ileum controls, ileum controls, and colon CD samples; Type E was ileum CD up-regulated genes (n = 3) and Type F was ileum CD down-regulated genes (n = 22). Wilcoxon signed-rank test and Shapiro test with t-test were used for comparison of two groups with p-value of 0.05 considered statistically significant. P-value of each group is displayed. NM, Normal (control).

a

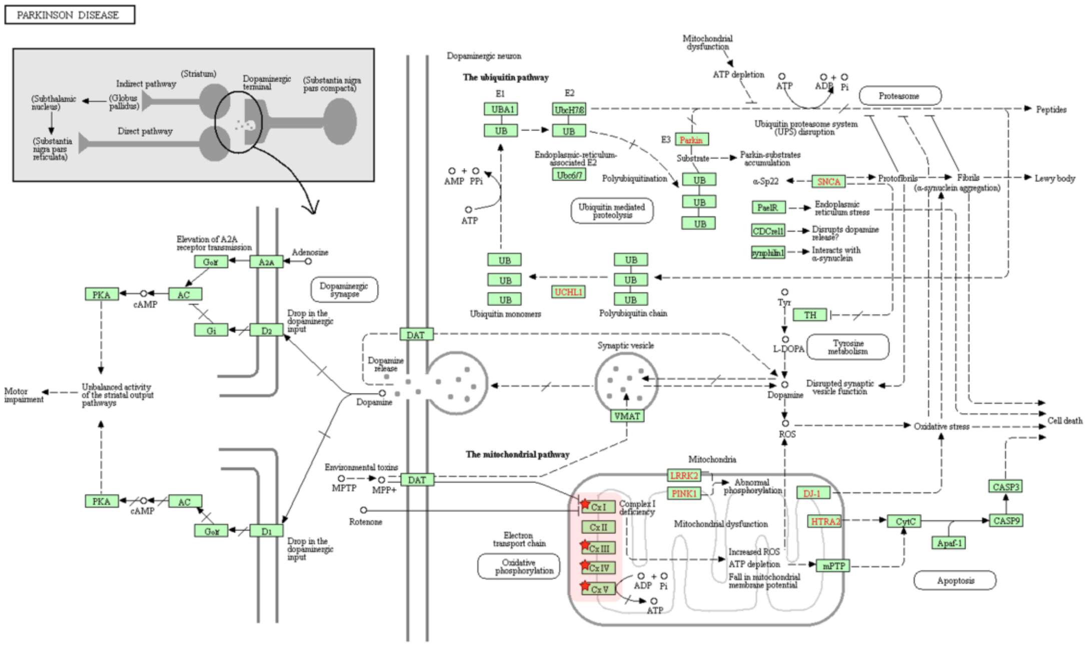

b

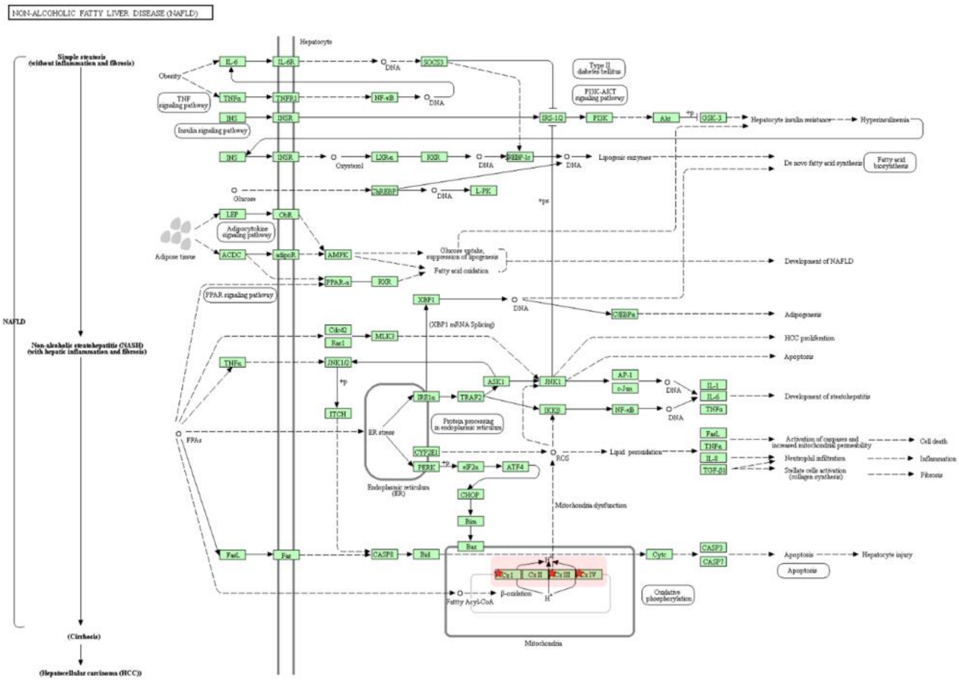

Fig. S8. Two major disease of KEGG Pathway from CCGs

(a) Parkinson Disease. **(b)** Non-Alcoholic Fatty Liver Disease.
KEGG pathway map05012 and map04932 are mined here from http://www.kegg.jp/kegg/kegg1.html.

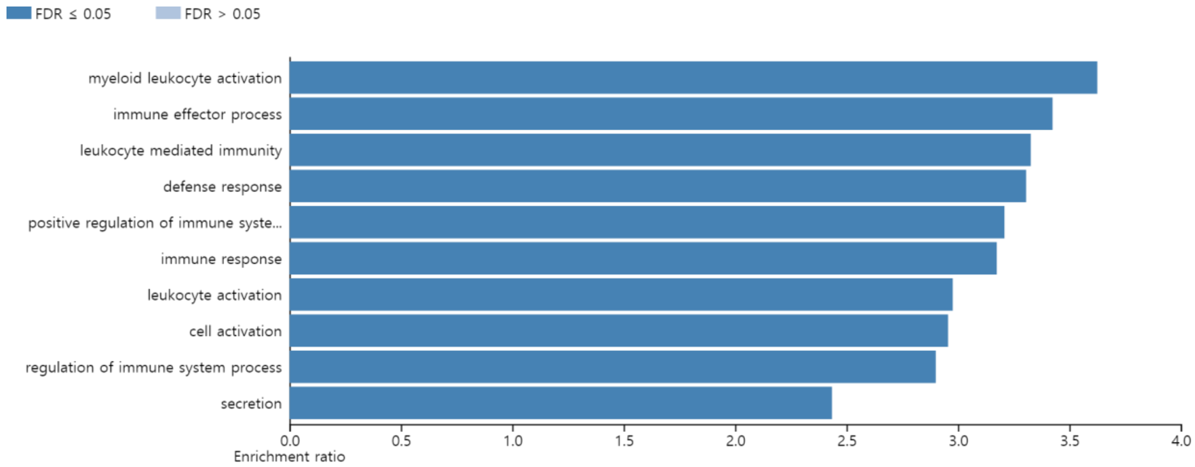

Fig. S9. Bar chart displaying biological function category from Gene ontology analysis of common genes (n=471) for Colonic CD and Terminal-Ileal CD

Colonic CD and Termianl-ileal CD share genes with the function of immune response. The x-axes represent enrichment ratio.

a

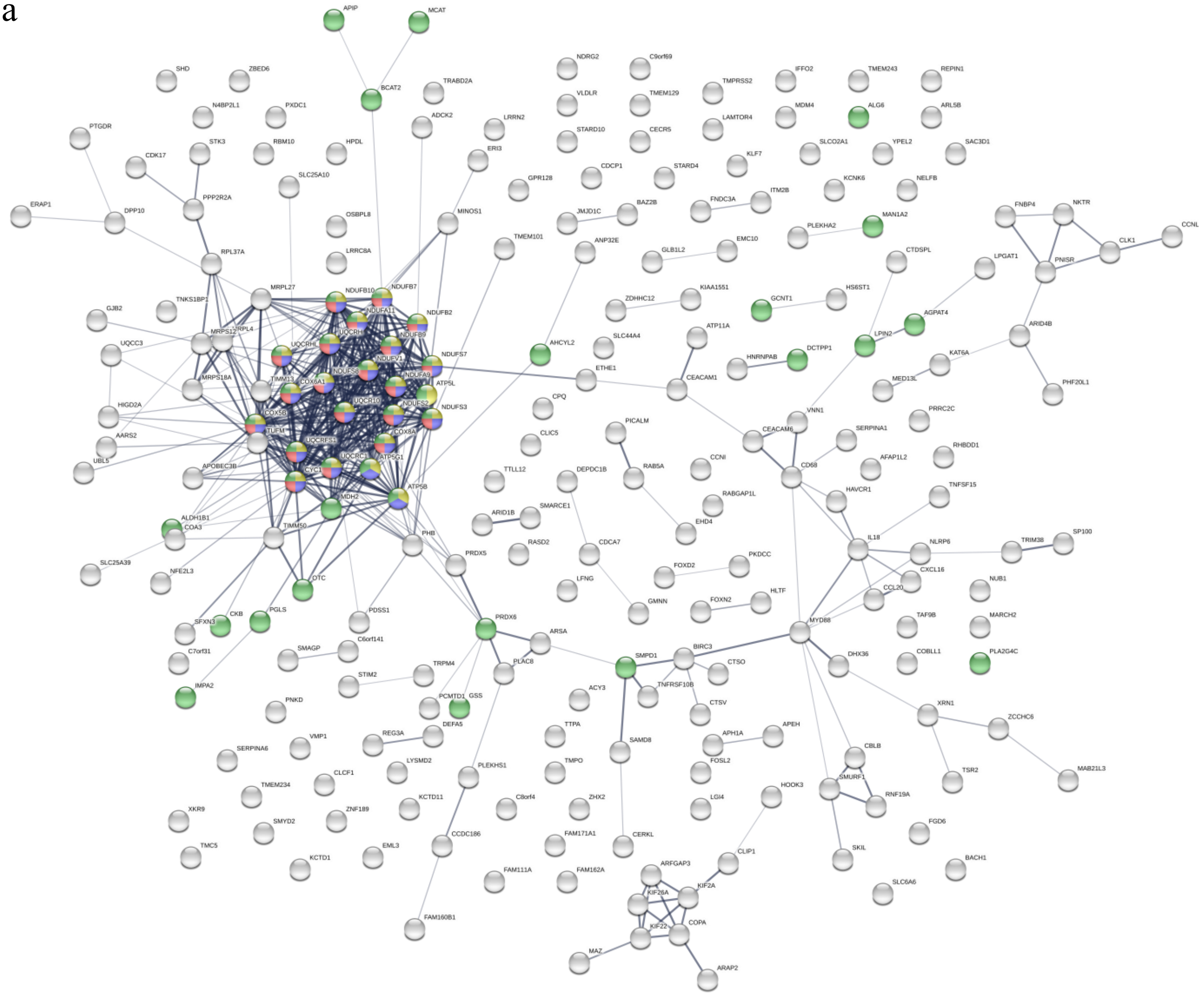
APIP
MCAT
BCAT2
TRABD2A
SHD
ZBED6
N4BP2L1
PXDC1
PTGDR
CDK17
STK3
RBM10
HPDL
ADCK2
LRRN2
ERI3
SLC25A10
PPP2R2A
ERAP1
DPP10
OSBPL8
MINOS1
LRRC8A
RPL37A
TMEM101
NDRG2
C9orf69
VLDLR
TMEM129
STARD10
CECR5
GPR128
CDCP1
STARD4
JMJD1C
BAZ2B
ANP32E
GLB1L2
TMPRSS2
IFFO2
TMEM243
REPIN1
LAMTOR4
MDM4
ALG6
ARL5B
KLF7
SLCO2A1
YPEL2
SAC3D1
ITM2B
KCNK6
NELFB
FNDC3A
PLEKHA2
MAN1A2
EMC10
CTDSPL
FNBP4
NKTR
CCNL
CLK1
PNISR
LPGAT1
ZDHHC12
KIAA1551
GCNT1
HS6ST1
AGPAT4
ARID4B
SLC44A4
ATP11A
LPIN2
KAT6A
HNRNPAB
DCTPP1
AHCYL2
ETHE1
CEACAM1
MED13L
PHF20L1
MRPL27
NDUFB10
NDUFB7
NDUFA11
NDUFB2
UQCRH
NDUFB9
TNKS1BP1
GJB2
MRPS12
RPL4
UQCRHL
NDUFV1
NDUFS7
UQCC3
NDUFS8
NDUFA9
TIMM13
COX6A1
ATP5L
MRPS18A
UQCR10
NDUFS2
NDUFS3
HIGD2A
COX5B
TUFM
COX8A
AARS2
UQCRFS1
UBL5
UQCRC1
ATP5G1
APOBEC3B
CYC1
ATP5B
MDH2
ALDH1B1
COA3
TIMM50
PHB
PRDX5
SLC25A39
NFE2L3
OTC
SFXN3
CKB
PGLS
PDSS1
PRDX6
C7orf31
SMAGP
C6orf141
ARSA
TRPM4
PLAC8
STIM2
IMPA2
PCMTD1
GSS
PNKD
DEFA5
SERPINA6
VMP1
REG3A
LYSMD2
PLEKHS1
CLCF1
TMEM234
KCTD11
XKR9
ZNF189
SMYD2
C8orf4
TMC5
KCTD1
EML3
CCDC186
FAM160B1
FAM111A
CPQ
CLIC5
PICALM
RAB5A
TTLL12
DEPDC1B
ARID1B
SMARCE1
RASD2
CDCA7
LFNG
GMNN
CCNI
RABGAP1L
EHD4
FOXD2
PKDCC
FOXN2
HLTF
SMPD1
BIRC3
CTSO
TNFRSF10B
CTSV
ACY3
TTPA
SAMD8
APH1A
APEH
FOSL2
TMPO
ZHX2
LGI4
FAM171A1
CERKL
FAM162A
HOOK3
CLIP1
ARFGAP3
KIF2A
KIF26A
KIF22
COPA
MAZ
ARAP2
VNN1
SERPINA1
PRRC2C
CEACAM6
RHBDD1
CD68
AFAP1L2
HAVCR1
TNFSF15
IL18
NLRP6
TRIM38
SP100
CXCL16
CCL20
NUB1
MYD88
TAF9B
MARCH2
DHX36
COBLL1
PLA2G4C
XRN1
ZCCHC6
CBLB
SMURF1
RNF19A
TSR2
MAB21L3
FGD6
SKIL
BACH1
SLC6A6

b

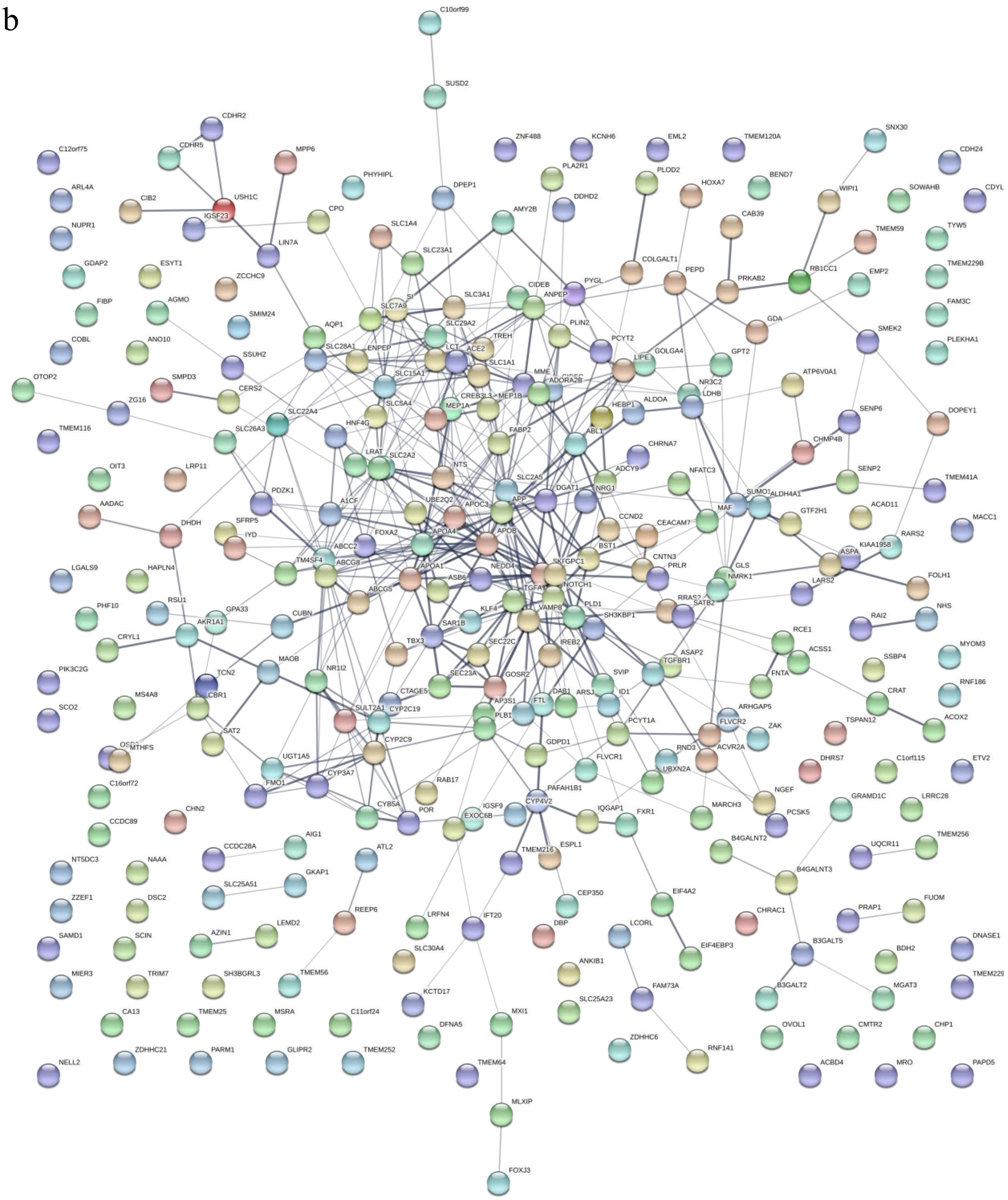

Fig. S10. Protein-protein interaction network for CCGs (n=240) and ICGs (n=310)

The string graph indicates genes with at least one interaction at the additional requirement of a log fold change >1·5, in which line width shows the strength of interaction. (a) Colonic CD genes (CCGs). Coloured circle indicates each KEGG pathway; blue: PD; red: NAFLD; yellow: oxidative phosphorylation; green: metabolic pathways. (b) Terminal-leal CD genes (ICGs).

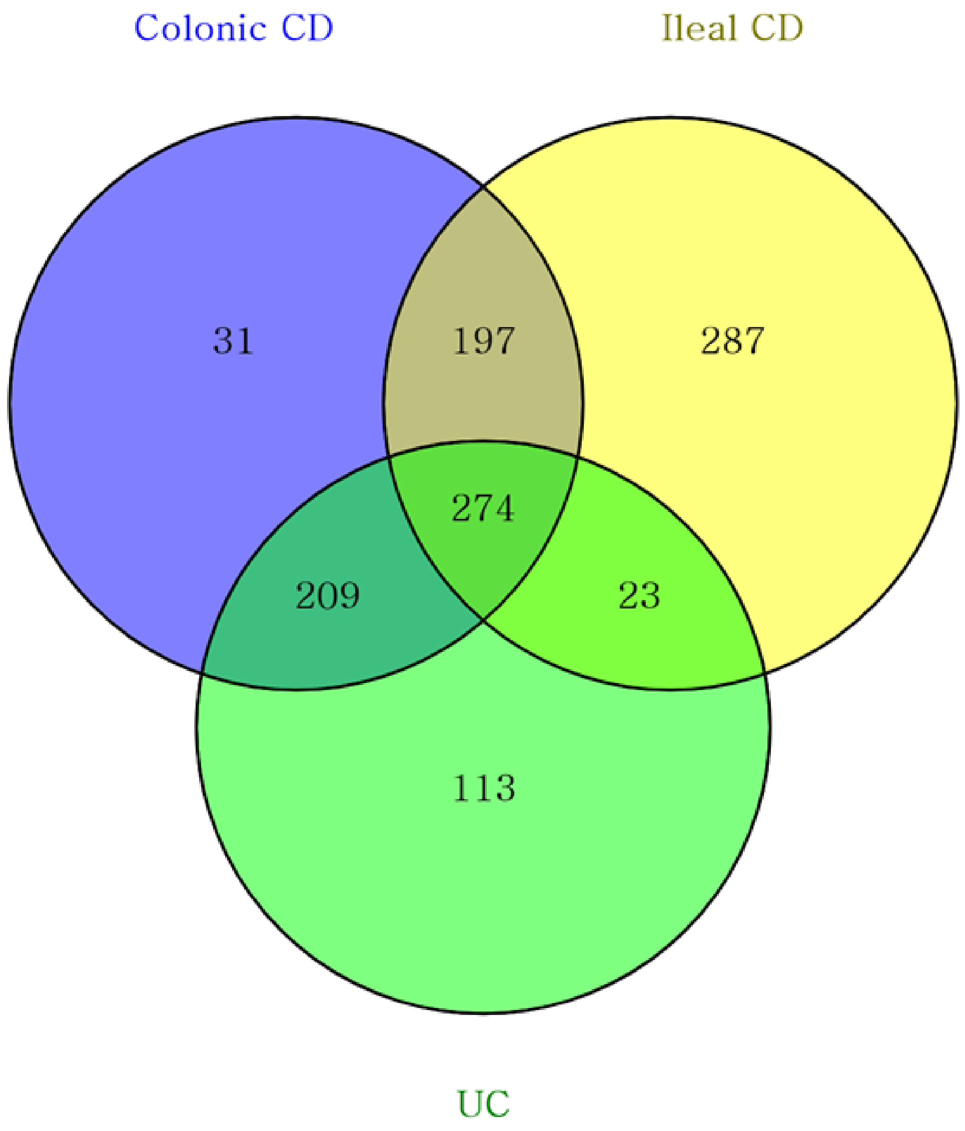

Fig. S11. A **venn diagram illustrating differentially expressed genes in colonic CD, ileal CD, and UC.**

Colonic CD genes [n=240] , ileal CD genes [n= 310], and UC genes [n= 619] were displayed in a venn diagram. Most UC genes were shared in CCGs [n=209] and CD common genes [n=274].

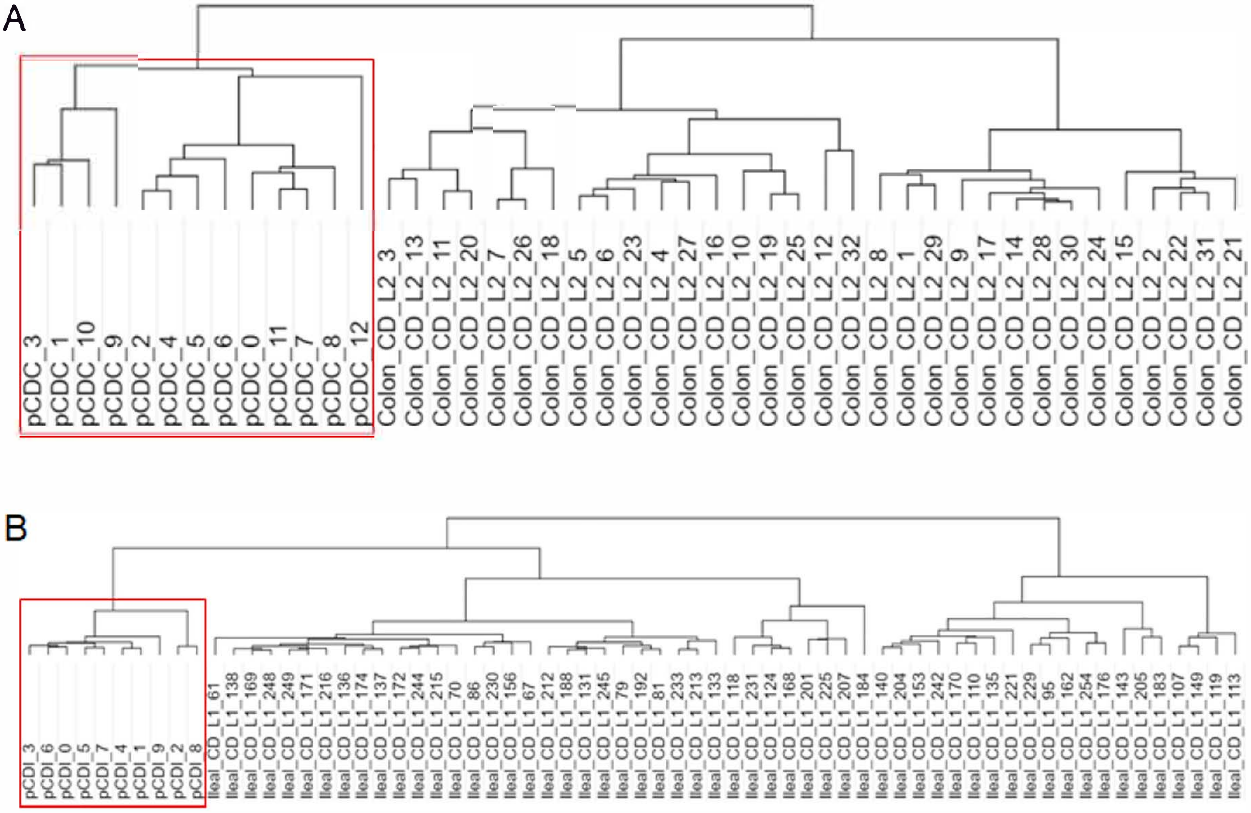

Fig. S12. Dendrogram of CD patients from different cohorts.

(A) The clustering result of patients with colonic CD. It is largely divided into two groups, and patients with colonic CD used in this sutdy belong to the left group in red box. (B) The clustering results of patients with ileal CD. It is largely divided into three groups, and although the ileal CD patients used in this study were clustered with the partial group of the independent cohort samples, they were still clearly distinguished by cohorts.
